# Transforming insect population control with precision guided sterile males with demonstration in flies

**DOI:** 10.1038/s41467-018-07964-7

**Published:** 2019-01-08

**Authors:** Nikolay P. Kandul, Junru Liu, Hector M. Sanchez C., Sean L. Wu, John M. Marshall, Omar S. Akbari

**Affiliations:** 1 Division of Biological Sciences, Section of Cell and Developmental Biology, University of California, San Diego, La Jolla, CA 92093 California USA; 2 Division of Biostatistics and Epidemiology, School of Public Health, University of California, Berkeley, CA 94720 California USA; 3 Tata Institute for Genetics and Society, University of California, San Diego, La Jolla, CA 92093 California USA

**Keywords:** Synthetic biology, CRISPR-Cas9 genome editing

## Abstract

The sterile insect technique (SIT) is an environmentally safe and proven technology to suppress wild populations. To further advance its utility, a novel CRISPR-based technology termed precision guided SIT (pgSIT) is described. PgSIT mechanistically relies on a dominant genetic technology that enables simultaneous sexing and sterilization, facilitating the release of eggs into the environment ensuring only sterile adult males emerge. Importantly, for field applications, the release of eggs will eliminate burdens of manually sexing and sterilizing males, thereby reducing overall effort and increasing scalability. Here, to demonstrate efficacy, we systematically engineer multiple pgSIT systems in *Drosophila* which consistently give rise to 100% sterile males. Importantly, we demonstrate that pgSIT-generated sterile males are fit and competitive. Using mathematical models, we predict pgSIT will induce substantially greater population suppression than can be achieved by currently-available self-limiting suppression technologies. Taken together, pgSIT offers to potentially transform our ability to control insect agricultural pests and disease vectors.

## Abstract (web summary)

Sterile Insect Technique (SIT) is used to suppress wild populations. Here the authors integrate CRISPR-based technology and SIT to develop a precision guided SIT (pgSIT), and demonstrate its proof-of-principle by generating 100% sterile males.

## Introduction

CRISPR-based genome editing has revolutionized the capacity for precise genome manipulations in nearly every organism studied (reviewed in ref. ^1^). For example, recently, it has been used to develop extremely efficient homing-based gene drives that can bias Mendelian inheritance rates with up to 99% efficiency in many animals including flies, mosquitoes, and mice^2–5^, revolutionizing an entire new field termed Active Genetics^6^. While these innovative technologies bear the potential to provide worldwide solutions to combat vector-borne diseases, improve agriculture, and control invasive species, ongoing discussions are underway to define the mechanisms of governance to ensure that the technology is ethically, and safely, developed and implemented^7–9^. Notwithstanding, current drive designs are limited by the rapid evolution of resistance^10^, and therefore future research is necessary to develop drives that can limit and overcome evolved resistance^11,12^. While these discussions and developments are advancing, given the precision, simplicity, and efficiency of CRISPR, we aimed to develop a novel, safe, and controllable, noninvasive, CRISPR-based genetic technology that could be transferred across species and implemented worldwide in the short term to combat wild populations.

Coined independently by Serebrovskii, Vanderplank, and Knipling, mass production and release of sterile males, known as the sterile insect technique (SIT), has historically been used to control, and eradicate, insect pest populations dating back to the mid-1930s^13–17^. Traditional methodologies have relied on DNA-damaging agents for sterilization, substantially reducing overall fitness and mating competitiveness of released males. To overcome these limitations, microbe-mediated infertility techniques such as *Wolbachia*-based incompatible insect technique (IIT)^18,19^, and modern genetic SIT-like systems such as the release of insects carrying a dominant lethal (RIDL)^20^, and other methodologies to release fertile males that genetically kill females such as female-specific RIDL (fsRIDL)^21^, and autosomal-linked X-chromosome shredders^22^ have been developed (reviewed in ref. ^23^). While these first-generation genetic SIT technologies represent significant advances, each approach has disadvantages. For example, IIT strictly requires no infected females be released which is impossible to achieve in the field, and the use of tetracycline known to ablate the microbiota^24^ compromises the fitness of RIDL/fsRIDL males, and X-chromosome shredders can in principle only be developed in species with heterogametic sex chromosomes, thereby limiting wide applicability to other species. Therefore, it would be logistically advantageous to employ more efficient SIT-based technologies that could simultaneously and efficiently sex-sort and sterilize males without significantly compromising their fitness, to date such optimal genetic technologies do not exist.

**Fig. 1:**
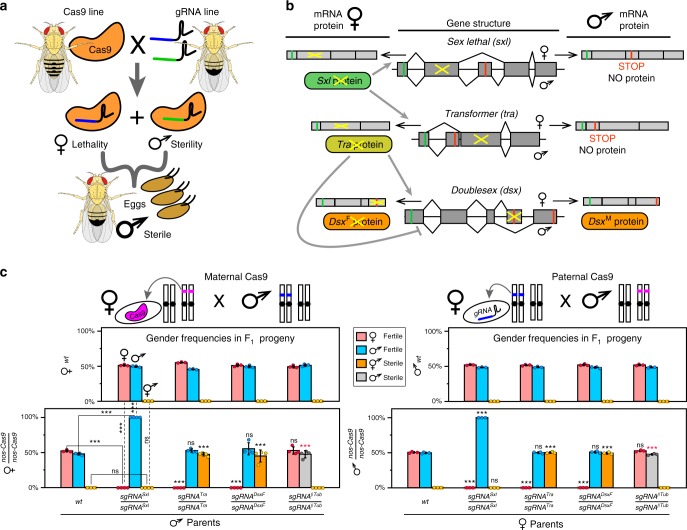
Precision guided sterile insect technique (pgSIT), an assessment of gene targets with single guide RNAs (sgRNAs). **a** A schematic of pgSIT utilizing two components of the binary CRISPR/Cas9 system, Cas9 and gRNAs, maintained as separated homozygous lines, their cross results in simultaneous knockouts of a gene required for female viability and a gene required for male fertility resulting in survival of only F_1_ sterile males. **b** A schematic of sex-specific alternative splicing in *sxl*, *tra,* and *dsx* regulated by female expression of Sxl and Tra proteins (gray lines) (modified from ref. ^68^). Disruption of female-specific exons of key sex-determination genes, *sxl*, *tra*, and *dsx*, disrupts female development. PgSIT exon targets indicated by yellow crosses. **c** Bar graphs of average gender frequencies in F_1_ progeny. Two top panels depict gender frequencies from bidirectional control crosses of homozygous sgRNA lines to wild type (*wt*), indicating that both fertile females and males (♀ and ♂) are present at similar ratios, but no sterile intersexes (⚥) were identified. Bottom two panels show gender frequencies from crosses of homozygous *nanos-Cas9* (*nos-Cas9*) to *wt* (control) and four homozygous sgRNA lines (experiment). Independent of maternal or paternal Cas9 inheritance, 100% of trans-heterozygous *sgRNA*^*Sxl*^ females were lethal, 100% of trans-heterozygous *sgRNA*^*Tra*^ and *sgRNA*^*DsxF*^ females were masculinized into sterile intersexes (⚥), and 100% of trans-heterozygous *sgRNA*^*βTu*^ males were sterile. Gender frequencies and fertility in trans-heterozygotes were compared to those in the corresponding progeny of control crosses with *nos-Cas9* (solid lines) or *sgRNAs* (dashed lines) and *wt* flies. Bars represent means ± SD for three/four independent groups of parental flies. *P>*0.001*** by a *t* test assuming unequal variance (black *) or, for male sterilization by Pearson’s chi-squared test for contingency tables (red *)

Here, we develop a next-generation highly efficient precision guided SIT (pgSIT) technology that can be deployed as eggs which exclusively give rise to sterile males. PgSIT functions by exploiting the precision and accuracy of CRISPR to simultaneously disrupt genes essential for female viability and male fertility. It utilizes a simple breeding scheme requiring two homozygous strains, one expressing Cas9 and the other expressing double-guide RNAs (*dgRNAs*). A single mating between these strains mechanistically results in synchronous RNA-guided dominant biallelic knockouts of both target genes throughout development, resulting in non-Mendelian complete penetrance of desired phenotypes in all progeny by converting recessive phenotypes into dominant phenotypes in a single generation (Fig. 1a). We demonstrate that pgSIT is extremely robust at genetically sexing and simultaneously sterilizing the resulting progeny reproducibly with 100% efficiency. Moreover, we show that pgSIT males are fit and can successfully compete for mates. Taken together, pgSIT offers to lead far superior population suppression over existing approaches, thereby revolutionizing SIT-mediated control of wild populations.

## Results

### Lethality and masculinization in females and male infertility

To engineer pgSIT, we first generated single-guide RNA (sgRNA) and spCas9 (Cas9 from hereon) expressing lines in *Drosophila*. In total, nine homozygous sgRNA lines were developed to target genes essential for female viability, or genes important for male fertility. For female viability, these genes included sex-specifically alternatively spliced sex-determination genes including s*ex lethal (Sxl*, two separate transgenic lines—*sgRNA*^*Sxl*^, s*gRNA*^*Sxl-B*^*)*, *transformer (tra*, two separate lines—*sgRNA*^*Tra*^, *sgRNA*^*Tra-B*^), or *doublesex* (*dsxF*, *sgRNA*^*DsxF*^*)* (Fig. 1b)^25–28^. To disrupt male fertility, we targeted genes active during spermatogenesis, such as *βTubulin 85D* (*βTub*, *sgRNA*^*βTub*^)^29^, *fuzzy onions (fzo*, *sgRNA*^*Fzo*^*)*^30^, *protamine A (ProtA*, *sgRNA*^*ProtA*^*)*^31^, or *spermatocyte arrest (sa*, *sgRNA*^*Sa*^*)*^32^ (Supplementary Fig. 1). To promote robust Cas9 expression, we established three homozygous Cas9-expressing lines under control of two strong predominantly germline-specific promoters, including *nanos (nos-Cas9)* or *vasa* (*vas-Cas9*)^33,34^, and one ubiquitous promoter to enable robust expression in both somatic and germline tissues during nearly all developmental life stages, *Ubiquitin 63E* (*Ubi-Cas9*)^35^(Supplementary Fig. 2). Downstream (3ʹ) to the promoter-driven Cas9, we included a self-cleaving T2A peptide and eGFP coding sequence, together serving as a visual indicator of promoter activity^36^ (Supplementary Figs. 1, 2).

To assess the genetic activity of the sgRNA lines, we crossed each strain to *nos-Cas9*, and examined the resulting trans-heterozygous F_1_ progeny. From these crosses, 4/9 of the sgRNAs, including *sgRNA*^*Sxl*^, *sgRNA*^*Tra*^, *sgRNA*^*DsxF*^, and *sgRNA*^*βTub*^, displayed expected phenotypes with complete penetrance and were subjected to further characterization. To further evaluate these four sgRNAs, we bidirectionally crossed each to wild type (*wt*) (replicate number of crosses between 10♀ and 10♂, *N* = 24; progeny number, *n* = 3519), or to homozygous *nos-Cas9* (*N* = 28, *n* = 3628; Supplementary Table 1). As expected, the *wt* crosses produced no significant gender ratio deviations or compromised fertility (*N* = 30, *n* = 4371) (Fig. 1c; Supplementary Table 1). Interestingly, however, regardless of whether *nos-Cas9* was maternally or paternally inherited, all F_1_ trans-heterozygotes inheriting *sgRNA*^*Sxl*^ were 100% male (*N* = 7, *n* = 540), and 100% of trans-heterozygous females inheriting *sgRNA*^*Tra*^ or *sgRNA*^*DsxF*^ were converted into sterile masculinized intersexes unable to oviposit eggs (*N* = 14, *n* = 942), and 100% of *sgRNA*^*βTub*^ trans-heterozygous males were sterile (*N* = 7, *n* = 517; Fig. 1c; Supplementary Table 1). These phenotypes were moleculary explored at the targeted genetic loci, and as expected, we discovered that all sequenced flies (*n* = 16) had mosaic insertions/deletions (indels) precisely at the targeted loci (Supplementary Table 2).

### Creation of populations of 100% sterile males

**Fig. 2:**
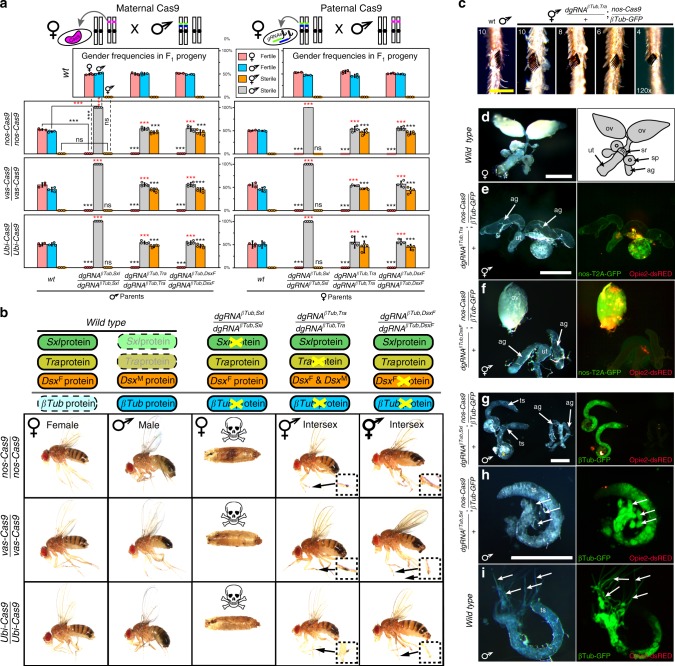
Development and characterization of multiple highly efficient pgSIT systems. **a** Gender (♀, ♂, and ⚥) frequencies of trans-heterozygous F_1_ progeny resulting from crosses between double gRNAs (*dsRNA*) and *Cas9* homozygous lines. Three dgRNAs, each targeting *sxl*, *tra*, or *dsx* combined with *βTub*, were bidirectionally crossed with three *Cas9* lines driven by *nanos* (*nos*), *vasa* (*vas*), and *Ubiquitin-63E* (*Ubi*) promoters and were sufficient to ensure complete penetrance of both female lethality/masculinization and male sterility in each reciprocal cross (Supplementary Figs. 1, 2). Gender frequencies and fertility in trans-heterozygotes were compared with those in the corresponding progeny of control crosses with *Cas9* (bar groups to the left, solid lines) or *dgRNAs* (top panels, dashed lines) and *wt* flies. Bars represent means ± SD for three/four independent groups of parental flies. *P>*0.01**, *P>*0.001*** by a *t* test assuming unequal variance (black *) or, for male sterilization by Pearson’s chi-squared test for contingency tables (red *). **b** Order of targeted gene in the sex-determination pathway (top) and the corresponding knockout phenotype in progeny. Phenotypes of *dgRNAs* directed knockouts and intersex morphology in comparison to *wt* females and males. *βTub*, *Sxl* double-knockout females perish during pupal stages (Supplementary Fig. 3). *dgRNA*^*βTub,Tra*^*/+*; *nos-Cas9/+*intersexes, but not *dgRNA*^*βTub,DsxF*^*/+*; *nos-Cas9/+ *intersexes, had sex combs, magnified inside inserts. **c** Variable expressivity of the number of sex comb bristles in *βTub*, *Tra* double-knockout intersexes. Scale bar shows 100 µm. **d** Internal reproductive organs in *wt* females: two ovaries (*ov*), seminal receptacle (*sr*), double spermatheca (s*p*), two accessory glands (*ag*), and uterus (ut). **e** Many *dgRNA*^*βTub,Tra*^*/+*; *nos-Cas9/+ *intersexes had one rudimentary ovary, and organs that resembled male accessory glands. **f** Many *dgRNA*^*βTub,DsxF*^*/+*; *nos-Cas9/+ *intersexes developed only a single ovary often times not connected with an oviduct and had organs that resembled male-specific accessory glands. **g**–**h** Male internal reproductive system in *dgRNA*^*βTub,Sxl*^*/+*; *nos-Cas9/+* male. In comparison with *wt* testis **i**, elongated cysts with maturing spermatids were not found in the *dgRNA*^*βTub,Sxl*^*/+*; *nos-Cas9/+* testis (**h**, **i**: arrows). Scale bars correspond to 500 µm

The goal of pgSIT is to disrupt genes essential for male fertility and female viability simultaneously to ensure that all surviving F_1_ offspring are sterile males. To achieve this feat, leveraging results described above, we generated three additional homozygous strains expressing multiplexed double gRNA (*dgRNA*) combinations, including *dgRNA*^*βTub,Sxl*^, *dgRNA*^*βTub,Tra*^, and *dgRNA*^*βTub,DsxF*^ (Supplementary Fig. 1). To genetically assess the activity of these pgSIT strains, we bidirectionally crossed each line to *wt* or homozygous Cas9 (either *nos-Cas9*, *vas-Cas9*, or *Ubi-Cas9)*. As expected, the *wt* crosses produced no significant gender deviations or compromised fertility (*N* = 36, *n* = 5747; Fig. 2a; Supplementary Table 3). Interestingly, however, the crosses between *dgRNA*^*βTub,Sxl*^ with each Cas9 strain resulted in 100% female lethality due to disruption of *sxl*, in addition to 100% male sterility due to simultaneous disruption of *βTub* (*N* = 24, *n* = 2521; Supplementary Table 3). Moreover, 100% females from crosses between each Cas9 strain and *dgRNA*^*βTub,Tra*^ (*N* = 24, *n* = 1697) or *dgRNA*^*βTub,DsxF*^ (*N* = 24, *n* = 1791) were masculinized into sterile intersexes due to disruption of either *tra* or *dsx*, and 100% male offspring were sterile due to simultaneous disruption of *βTub* (*N* = 48, *n* = 4231). These findings demonstrate that the ability to form highly active Cas9-gRNA complexes was not saturated by dgRNAs, and the pgSIT approach works reproducibly with unprecedented efficiency (Fig. 2a, b; Supplementary Table 3).

In terms of phenotypes, we found that the 100% of the *dgRNA*^*βTub,Sxl*^ knockout females perished during preadult stages with the majority dying during pupal transition (Supplementary Fig. 3; Supplementary Tables 4, 5). For intersex phenotypes, fertility was always compromised; however, variable expressivity was observed as the extent of anatomical masculinization varied between individuals and was more pronounced in the *dgRNA*^*βTub,Tra*^ knockout as compared with the *dgRNA*^*βTub,DsxF*^ (Fig. 2b; Supplementary Table 4). For example, *dgRNA*^*βTub,Tra*^ knockout intersexes had sexcombs with variable bristle numbers (Fig. 2b, c; Supplementary Table 6), and rarely developed more than one rudimentary ovary (Fig. 2d, e; Supplementary Table 6). Moreover, molecularly the *dgRNA*^*βTub,Tra*^ knockout intersexes expressed both female- and male-specific alternative splice variants of *dsx* gene (Supplementary Fig. 4), presumably due to the absence of Tra which is important for inhibiting the male-specific and promoting the female-specific alternative splicing of *dsx*^37^. In contrast, the *dgRNA*^*βTub,DsxF*^ knockout intersexes were not observed to develop sexcombs, and some intersexes had normal ovaries enabling them to become gravid, although unable to oviposit (Fig. 2b, f; Supplementary Table 6).

In regard to male infertility phenotypes, to visualize the anatomy of testes and developing spermatids, in the F_1_ sterile males, we generated a transgenic line expressing eGFP under control from the *βTub85D-promoter (βTub*-*GFP)* to fluorescently label the testes and sperm (Supplementary Fig. 1), and introgressed it with the *dgRNA* strains. When introgressed with homozygous *nos-Cas9*, the trans-heterozygous *dgRNA*^*βTub,Sxl*^*/+*; *βTub-GFP*/*nos-Cas9* F_1_ sterile males had fully developed coiled testes and accessory glands (Fig. 2g); however, spermatid development was completely disrupted with phenotypes consistent with previous *βTub* disruption reports^29^. For example, only round cysts and early spermatocytes were identified in the testes of sterile males marked with GFP (Fig. 2h), while *wt* testes had robust GFP-labeled cysts with elongated late spermatids (Fig. 2i). Moreover, given that the *βTub*-*GFP* labels testes/sperm, this tool enabled us to explore the internal anatomy of reproductive systems in intersexes to search for putative male testes-like structures. Although no GFP-positive testes were identified in either *dgRNA*^*βTub,Tra*^ or *gRNA*^*βTub,DsxF*^ knockout intersexes (*n* > 20, Supplementary Table 6), paired putative male accessory gland-like organs were present in both intersex types (Fig. 2d, e–g). Finally, to confirm the molecular changes that resulted in knockout phenotypes, we sequenced both targeted loci from individual F_1_ flies. As expected, compared with the control flies (*n* = 32), each examined double-knockout fly (*n* = 20) had mosaic indels precisely at the cleavage sites that prevented sequencing through both ends of PCR amplicons (Supplementary Figs. 5, 6; Supplementary Table 2).

### Complete penetrance resulting from zygotic expression

**Fig. 3:**
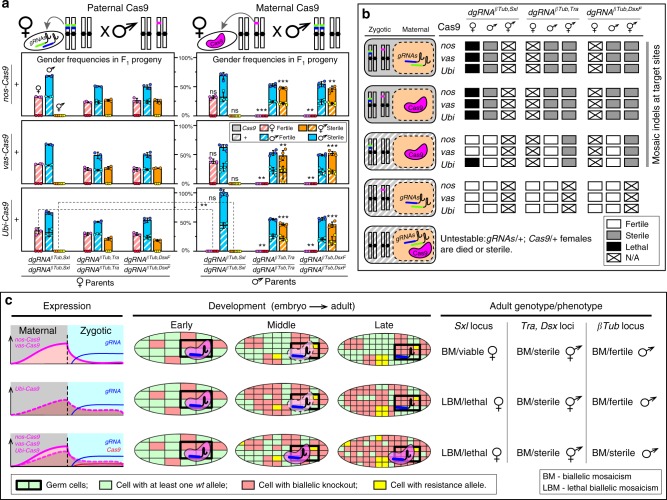
Zygotic expression of CRISPR/Cas9 components ensures 100% penetrance. **a** Genetic quantification of dominant effect by maternal loading of Cas9 protein. Genotypes, gender frequencies, and fertility of progeny flies generated by reciprocal crosses between homozygous *dgRNAs* and heterozygous *Cas9* flies. Bars represent means ± SD for three/four independent groups of parental flies. Gender frequencies from crosses with heterozygous maternal *Cas9* were compared with those from the corresponding crosses with heterozygous paternal *Cas9*. *P* *>* 0.01**, *P* *>* 0.001*** by a *t* test assuming unequal variance. Solid bars indicate inheritance of *Cas9* as a gene, while striped bars indicate inheritance of + allele. **b** Combinations of genotypes and maternal/zygotic contributions in embryos, and their penetrance. **c** Accumulation of high levels of biallelic mosaicism (BM) throughout development leads to the loss of gene function at the organismic level and ensures complete penetrance of induced phenotypes: lethality (lethal biallelic mosaicism (LBM)), female masculinization, or male sterility. Complementation of gene function in some cells by uncleaved *wt* alleles, and resistance alleles generated by NHEJ, are not sufficient to rescue the induced phenotype at the organismic level and therefore 100% of trans-heterozygous progeny have the induced phenotypes. Boxes get smaller and more abundant as cells divide

Maternal deposition of Cas9/gRNA complexes into developing embryos is sufficient to ensure non-Mendelian inheritance of mutations in receiving progeny, even if those progenies do not genetically inherit the genes encoding the editing components, and this phenomenon is known as a dominant maternal effect^38^. To extend this work, we aimed to test whether paternal inheritance of one of the core components (i.e., *Cas9* or *dgRNA*), combined with maternal deposition of the compatible component, would be sufficient to generate F_1_ knockout phenotypes. For the first combination, matings between homozygous Cas9 fathers and heterozygous dgRNA-expressing mothers were not sufficient to induce mutations (*n* = 12), or knockout phenotypes (*N* = 6, *n* = 252), in F_1_ progeny that did not inherit the *dgRNAs* as a gene, presumably a result of a short dgRNA half-life in the absence of Cas9 during maternal deposition (Fig. 3a, b; Supplementary Table 2). Moreover, matings between heterozygous Cas9 fathers and homozygous dgRNA-expressing mothers resulted in male sterility and female lethality/masculinization phenotypes in all trans-heterozygous F_1_ progeny that inherited the *Cas9* gene (*N* = 27, *n* = 2191), while all F_1_ progeny that inherited only the dgRNA-encoding genes maintained normal features (*N* = 27, *n* = 2640; Fig. 3a; Supplementary Table 7). More specifically, crosses between heterozygous Cas9 mothers and homozygous dgRNA-expressing fathers resulted in male sterility and female lethality/masculinization phenotypes in all trans-heterozygous F_1_ progeny (*N* = 36, *n* = 3019; Fig. 3a; Supplementary Table 7). Additionally, maternal contribution of Cas9 protein was sufficient to induce intersex phenotypes in progeny that did not receive the *Cas9* gene when targeting *tra* or *dsx* (*N* = 24, *n* = 782), demonstrating the dominant maternal effect (Fig. 3a). However, maternal contribution of Cas9 only by *Ubi-Cas9* (*N* = 4; *n* = 0, number of surviving females), but not *nos-Cas9* nor *vas-Cas9* (*N* = 8, *n* = 556), induced *dgRNA*^*βTub,Sxl*^*/+*; *+/+*  female lethality indicating that promoter strength likely plays an important role in mutation efficiency resulting from the maternal effect (Fig. 3a, b). Interestingly, despite the lack of lethality phenotypes in females receiving Cas9 protein maternally loaded from *nos-Cas9* and receiving the *dgRNA*^*βTub,Sxl*^ gene, these surviving females had mosaic indels at the *Sxl* locus (*n* = 2, Supplementary Table 2; Fig. 3b). Similarly, all male progeny that inherited only the *dgRNA* genes (*N* = 36, *n* = 1490), and had maternally loaded Cas9 protein, were fertile regardless of Cas9 strain used (Fig. 3a), though each genotyped male (*n* = 6) had mosaic indels at the *βTub* locus (Supplementary Table 2; Fig. 3b). Taken together, these results indicate that paternal inheritance of gRNAs along with maternal deposition of Cas9 into developing embryos, in the absence of Cas9 inherited as a gene, was sufficient to induce detectable biallelic mosaicism, although penetrance was incomplete depending on the gene targeted (Fig. 3c).

### Fitness of pgSIT males is not compromised

**Fig. 4:**
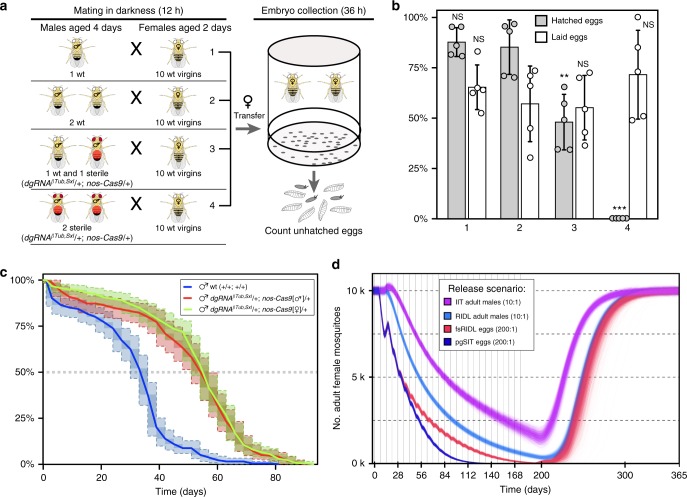
Competitiveness and longevity of pgSIT males and modeling data. **a** An experimental setup to estimate the mating competitiveness of *dgRNA*^*βTub,Sxl*^*/+; nos-Cas9/+* sterile males (marked with red) competing against *wt* males to secure matings with *wt* females. A mated female is resistant to the next mating for around 24 h^64,69^, and the mating success of sterile males was evaluated by fertility decrease (aka. increase of unhatched egg rate). **b** Bars graph percentages of laid and hatched eggs (Supplemental Table 8). The presence of one sterile male resulted in a significant decrease in female fertility (#3 vs. #2) that could not be accounted by removal of one *wt* male (#2 vs. #1). Bars represent means ± SD for five replicates. #3 to #2 and #1 *P* *>* 0.003**, *P* *>* 0.0001*** by a *t* test assuming unequal variance. **c** Survival curves of *wt* males (blue line) and two types of *dgRNA*^*βTub,Sxl*^*/+*; *nos-Cas9/+* sterile males, with paternal (red line) or maternal (green line) Cas9 inheritance. Survival curves show nonparametric maximum likelihood estimates for three male groups, along with 95% confidence intervals in light shade and dark shade for representational nonuniqueness. The *y*-axis shows the estimated survival percentage. Both types of pgSIT males lived significantly longer than *wt* males (*P* *<* 2.2^–16^), while no significant difference was found between two types of pgSIT males by Sun’s generalization of the log-rank test. **d** Model-predicted impact of releases of pgSIT eggs on *Aedes aegypti* mosquito population density with comparison to releases of *Wolbachia*-based incompatible insect technique (IIT), release of insects carrying a dominant lethal gene (RIDL), and female-specific RIDL (fsRIDL). Releases are carried out weekly over a 6-month period with release ratios (relative to wild adults) shown in the key. Model predictions were computed using 2000 realizations of the stochastic implementation of the MGDrivE simulation framework^43^ for a randomly mixing *Ae*. *aegypti* population of 10,000 adult females and model parameters described in Supplemental Table 10. Notably, pgSIT releases outcompete those of all other suppression technologies, showing the highest potential to eliminate the local population

We expected that precise knockouts of single genes required for female-specific viability and spermatid maturation would not significantly affect the overall fitness of pgSIT males. To assess the fitness of pgSIT males, we performed a mating competition assay (Fig. 4a) and estimated survival curves. We found that pgSIT-generated males were able to court, mate, and successfully compete with *wt* males. A reduced egg hatch rate of 47.9% ± 13.8% for one *wt* together with one pgSIT male vs. 85.1% ± 13.5% for two *wt* males (*N* = 5, *P* *>* 0.003 for a *t* test assuming unequal variance) or 87.6% ± 7.2% for one *wt* male (*N* = 5, *P* *>* 0.001; Fig. 4b; Supplementary Table 8) was consistent with a mating competitiveness of pgSIT males of 78% than that of *wt* males. Notably, we also discovered that longevity was not compromised in pgSIT males as compared with *wt* males (Fig. 4c). Because maternally deposited Cas9 is known to affect progeny phenotype^38^, two types of pgSIT males, one inherited paternal Cas9 and the other maternal Cas9, were considered separately. The median survival time for *wt* males was estimated as 32.3 ± 1.3 days (*N* = 5, *n* = 275); and median survival times of pgSIT males were 52.7 ± 1.6 days (*N* = 5, *n* = 220) or 53.7 ± 0.9 days (*N* = 5, *n* = 275) for males carrying paternal or maternal Cas9, respectively (Fig. 4c; Supplementary Table 9). Both types of pgSIT males survived significantly longer than *wt* males (*P* *<* 2.2^–16^ by Sun’s generalization of the log-rank test; Fig. 4c), while no significant difference was identified between two types of pgSIT males. Different median survival times were reported for *Drosophila* wild-type males, for example, 35.5 days^39^ and 57 days;^40,41^ breeding conditions, such as food composition, temperature, etc., are known to affect survival time. The median survival time of pgSIT males is similar to the longer survival times reported for *wt* males; thus, we can conservatively estimate that survival of pgSIT males was not reduced. Taken together, pgSIT males were able to compete for mates and survive for extended periods.

### PgSIT and current methods to suppress mosquito populations

Given the simplicity and consistency of generating sterile males (Fig. 1a), pgSIT could potentially be used in the future to mass produce and release eggs into the environment to suppress target populations. A potential application of pgSIT would be the suppression of populations of *Ae*. *aegypti*, the mosquito vector of dengue, Zika, and Chikungunya. *Ae*. *aegypti* has an obligatory diapause during the egg stage, and thus dessicated eggs can survive for extended periods^42^. To explore how the pgSIT approach may fare against currently available self-limiting suppression technologies—namely RIDL, fsRIDL, and IIT—we simulated release schemes for each of these technologies using the MGDrivE simulation framework^43^. This framework models the egg, larval, pupal, and adult mosquito life stages with overlapping generations, larval mortality increasing with larval density, and a mating structure in which females retain the genetic material of the adult male with whom they mate for the duration of their adult lifespan^43^. We consider releases into a randomly mixing population consisting of 10,000 adult females, with model and intervention parameters listed in Supplementary Table 10.

We simulated weekly releases of adult males for RIDL and IIT and eggs for fsRIDL and pgSIT over a 6-month period (Fig. 4d). Adult release ratios were ten adult RIDL/IIT males per wild adult, following the precedent of a field trial of *Ae*. *aegypti* RIDL mosquitoes in Brazil^44^, and egg release ratios were 200 eggs per wild adult, given that female *Ae*. *aegypti* produce ~20 eggs per day in temperate climates^45^. Results from these simulations suggest that systems for which eggs are released (pgSIT and fsRIDL) result in the most rapid population suppression in the first 3 weeks as released eggs quickly hatch as larvae and reduce the survival of fertile larvae as a consequence of density-dependent larval competition. The pgSIT approach shows the greatest suppression from the end of the first month on, and the greatest potential to eliminate the population during the release period. This is due to the higher mating competitiveness of pgSIT males (78% that of *wt* males) c.f. fsRIDL males (~5% that of *wt* males, based on RIDL field trials in the Cayman Islands^46^ and Brazil^44^), which becomes a dominant factor at low population densities when greater consumption of larval resources by released immature forms has less impact on suppression. Population suppression resulting from 10:1 releases of adult RIDL males trails that for releases of fsRIDL eggs by 2–3 weeks due to the delay in impact on density-dependent larval competition; but is similar in magnitude. Equivalent releases of adult IIT males are less impactful for the strategy we consider, in which male incompatibility is induced through *Wolbachia* infection and the chance of an unintended release of *Wolbachia*-infected females interfering with suppression is reduced through low-level irradiation^47,48^, resulting in the longevity of released IIT males being roughly halved^49^.

In summary, these results suggest that pgSIT has greater potential to eliminate local *Ae*. *aegypti* populations than currently available suppression technologies. The results also appear highly robust to variation in the lifespan and mating competitiveness of pgSIT adult males (Supplementary Fig. 7). For weekly releases of 200 pgSIT eggs per wild adult, simulations suggest a wide range of parameter values for which local *Ae*. *aegypti* elimination could be reliably achieved (male mating competitiveness >~25%, lifespan reduction <~75%). Elimination could also be reliably achieved for smaller releases of 100 pgSIT eggs per wild adult (male mating competitiveness > ~50%, lifespan reduction <~50%).

## Discussion

CRISPR has empowered the development of a novel system termed pgSIT to enable the release of eggs ensuring all progeny surviving to adulthood are sterile males—a feat never before possible. This is accomplished by using advanced molecular genetics to simultaneously sterilize males and eliminate females. Importantly, pgSIT relies exclusively on highly efficient CRISPR-mediated DNA cleavage, and NHEJ-based repair, and does not rely on homology-directed repair (HDR). Therefore, generation of resistance alleles that can curtail CRISPR-mediated gene drives^10^ does not limit the efficacy of pgSIT, as absolute disruption of *wt* alleles is not required to ensure complete penetrance of induced phenotypes when targeting essential genes, a phenomenon we term lethal biallelic mosaicism (Fig. 3c). Additionally, accumulation of resistance is unlikely to pose an issue for pgSIT since homozygous strains are raised separately and then mated to produce sterile males which do not generate viable progeny, limiting the selection pressure on the CRISPR target sites. Given that the role of pgSIT males is simply to seek out wild females, mate, and thereby reduce fecundity, natural genetic diversity in the wild is also not likely to pose a problem.

In terms of the underlying mechanism for pgSIT’s extreme efficiency, we determined that maternal and zygotic activity of the Cas9/gRNA complexes ensures continuous biallelic mosaicism of targeted alleles throughout development, resulting in complete penetrance of desired phenotypes, although variable expressivity was still observed depending on the gene targeted and on the timing and strength of the promoter-driving expression of Cas9 (Fig. 3c). Moreover, paternal inheritance of dgRNAs along with maternal deposition of Cas9 into developing embryos, in the absence of Cas9 inherited as a gene, was also sufficient to induce detectable biallelic mosaicism for all genes targeted (*βTub*, *dsx*, *tra*, and *sxl*), although penetrance was sometimes incomplete depending on the gene targeted. For example, maternal deposition of Cas9 alone was sufficient to induce intersex phenotypes (*dsx* and *tra*), however, it was insufficient to phenotypically ensure male sterility (*βTub)*, and depended on the strength of the promoter maternally depositing Cas9 to ensure female death (*sxl*) via lethal biallelic mosaicism (Fig. 3b, c). Taken together, these observations suggest that rates of biallelic mosaicism which ensure complete penetrance depend exclusively on whether components (i.e., Cas9 and gRNA) are inherited as genes or maternally deposited. Additionally, regardless of how the components are inherited, if rates of biallelic mosaicism are over a critical threshold, which is specific to each gene targeted, then complete penetrance can be achieved. Mechanistically, this technology demonstrates a fundamental advance in genetics by which somatic biallelic disruptions in essential genes, that previously conferred recessive phenotypes, get simultaneously converted by pgSIT in many somatic cells resulting in dominant fully penetrant phenotypes in a single generation.

Recently, Kyrou et al.^50^ described a CRISPR homing-based suppression drive that targeted *dsx* that also relied on biallelic mosaicism to ensure complete penetrance of a female sterility phenotype. Interestingly, in population cage experiments, their suppression drive resulted in complete collapse of two laboratory populations of *Anopheles gambiae*. While exciting, it is unclear how this drive will fare against massive diverse wild populations which will likely impose strong selection for appearance of resistance alleles that are predicted to rapidly prevent the spread of such a drive^51^. In fact, accumulated resistance poses a great obstacle to any homing-based gene drive; however, it may be possible to curtail this resistance by further exploiting lethal biallelic mosaicism as an added feature to all gene drives. For example, although this remains to be demonstrated, lethal biallelic mosaicism could also be exploited to engineer evolutionary stable gene drives which rely on toxin–antidote combinations to ensure that the drive is less prone to induce the rapid accumulation of resistant alleles. The design of such drives could rely on a linked toxin–antidote combination which could either incorporate homing (Supplementary Fig. 8a, b), or be mechanistically cleavage-only-based drives with no homing required (Supplementary Fig. 8c, d). For example, the mechanism of such drives could rely on Cas9-gRNA cleavage of an essential gene (as a toxin) induced by the gene drive insuring all progeny are killed via lethal biallelic mosaicism unless they inherit the drive that encodes a linked cleavage-resistant recorded copy of the targeted essential gene (as an antidote). Future efforts should focus on developing and testing these novel types of drives in model systems and later in vectors of disease.

The simplicity of the system provides a rationale for developing pgSIT in many insect species, including disease vectors and agricultural pests. Importantly, the technology does not rely on chromosome translocations, chemosterilants, irradiation, antibiotics, or bacterial infections, which can severely compromise the fitness and mating competitiveness of released sterile males. To implement pgSIT in disease vectors, many genes important for female viability and male fertility can be targeted. For example, given their functional conservation, *dsx*^52,53^ and *βTub*^54,55^ could initially be tested in mosquitoes. Alternatively, there are a plethora of other female/male-specific genes that could also be targeted^56,57^. Notwithstanding, while there are many genes to target, care must be taken in target gene selection to ensure minimal negative impacts on male fitness, male courtship behavior, and female sexual satiation. Moreover, given that highly efficient, genomically encoded Cas9-expressing strains have already been developed in major dengue and malaria disease vectors including *Ae*. *aegypti*^36^, *Anopheles gambiae*^5^, and *Anopheles stephensi*^4^, suggests that pgSIT may be trivial to develop in these species. To efficiently utilize pgSIT for mosquitoes, we envision the development of a rearing facility to propagate homozygous Cas9 and dgRNA-expressing strains separately. An automated workflow would also need to be implemented to sex-sort immature stages (e.g., Cas9 females with dgRNA males) and combine into cages for maturation, mating, and propagation of eggs. Sex sorting can be achieved in a number of ways including mechanical size separation, automated copas sex-sorting platform (Union Biometrica) combined with a genetic sexing strain, or automated robotic optical sorting and therefore should not be an insurmountable limitation (reviewed in refs. ^58,59^). It should be noted that pgSIT would be quite effective for the insect species which diapause during the egg stage, for example, *Ae*. *aegypti* and *Ae*. *albopictus*, to enable scalable egg accumulation for inundative releases. A single efficient pgSIT egg production facility could distribute pgSIT eggs to many remote field sites all over the world, where they could simply be hatched, reared, and released, reducing costs of building multiple production facilities, as well as eliminating the logistical burden of manual sex-sorting, sterilization, and releasing fragile adult males in each field location, thereby increasing scalability and efficiency, enabling broader wide-scale population suppression capacity (Supplementary Fig. 9).

Mathematical modeling of pgSIT alongside currently available self-limiting suppression technologies—RIDL, fsRIDL, and IIT—suggests that pgSIT has the highest potential to eliminate local *Ae*. *aegypti* populations and highlights the relative strengths of the pgSIT approach, even before the cost-effectiveness and scalability of egg releases are taken into account (Fig. 4d). Egg releases result in rapid population suppression from the outset, as hatching larvae consume resources that would otherwise be available to fertile larvae. A beneficial property shared by both pgSIT and fsRIDL is that all released eggs can result in hatching larvae, as female lethality occurs during embyo/larval stages, resulting in maximum consumption of larval resources by released immature forms. We predict pgSIT to achieve greater suppression than fsRIDL and RIDL, in their current forms, due to the substantially higher mating competitiveness (~78% that of *wt* males) c.f. RIDL males (~5% that of *wt* males) and the longer survival of pgSIT males. Mating competitiveness and longevity are dominant factors in achieving local elimination, as once initial suppression has been achieved, larval resources are abundant and hence greater consumption by released immature forms is less impactful.

Improving the mating competitiveness of RIDL males is conceivably an engineering problem hinging on reducing toxin leakage (Tet-Off) following rearing with tetracycline; however, the cause of such a large reduction in mating competitiveness is, to our knowledge, unclear. Regardless, pgSIT has an additional advantage over fsRIDL when it is preferred that introduced transgenes do not persist in the environment for more than a generation following their final release. Additional excitement for pgSIT stems from its potential to eliminate local *Ae*. *aegypti* populations for a wide range of lifespan and mating competitiveness parameter values (Supplementary Fig. 7c), suggesting some wiggle room when porting to other species. Simulations also suggest that pgSIT may be capable of eliminating local populations given smaller release ratios (Supplementary Fig. 7d–f). Combined with the feasibility and cost-effectiveness of mass rearing and release of pgSIT eggs, this points to a highly promising technology for the suppression of local populations of insect agricultural pests and disease vectors.

One limitation of all CRISPR-based technologies that potentially can also affect pgSIT is Cas9-based off-target cleavage^60^. In fact, ionizing radiation-induced random mutagenesis is the major cause of the sterility as well as the lower fitness of released males generated with classical SIT methods. That said, while pgSIT males may indeed harbor off-target genome modifications generated by Cas9/gRNA off-target activity, their numbers are likely low because the fitness of pgSIT males was not significantly affected. For example, pgSIT males were able to survive for extended periods and compete for mates (Fig. 4a–c). Taken together, these considerations suggest that pgSIT is an efficient and yet environmentally friendly technology for insect population control.

## Methods

### CRISPR target site design

To confer female lethality and male sterility, target sites for guide RNAs (gRNAs) were chosen inside female-specific exons of sex-determination genes, *Sex Lethal (Sxl)*, *Transformer (tra)*, and *Doublesex (dsx)*, and in male-specific genes, *βTubulin 85D* (*βTub*), *fuzzy onions* (*fzo)*, *Protamine A (ProA)*, and *spermatocyte arrest (sa)*, respectively. CHOPCHOP v2^61^ was used for choosing gRNA target sites from a specified sequence in *Drosophila* genome (dm6) to minimize the off-target cleavage. Due to the alternative splicing, functional Sxl and Tra proteins are produced only in *Drosophila* females^26,27^, while two versions of Dsx protein—female (Dsx^F^) or male (Dsx^M^)—are made each in the corresponding gender^28^ (Fig. 1b). The gRNA target for *βTub* was chosen in the vicinity to the *βTub85D*^*D*^ (*B2t*^*D*^) mutant allele^29^. Sequences of gRNA target sites are presented in Supplementary Fig. 1.

### Design and assembly of constructs

The Gibson enzymatic assembly method was used to build all constructs^62^. The previously described plasmid harboring the *SpCas9-T2A-GFP* with nuclear localization signals (NLS) flanking SpCas9 coding sequence and the *Opie2-dsRed* transformation marker was used to build *Drosophila* Cas9 constructs. This plasmid was used for *Ae*. *aegypti* transgenesis and had both piggyBac and an attB-docking sites (Addgene #100608)^36^. The *Ae*. *aegypti* promoter was removed from the plasmid by cutting at NotI and XhoI sites and replacing it with *Nanos* (*nos*), or *Ubiquitin-63E* (*Ubi*), or *Vasa* (*vas*) promoter (Supplementary Fig. 1). Promoter fragments were PCR amplified from *Drosophila* genomic DNA using the following primers: nos-F, nos-R, Ubi-F, Ubi-R, vas-F, and vas-F (Supplementary Table 11). To generate constructs with a single gRNA, *Drosophila* U6-3 promoter and guide RNA with a target, scaffold, and terminator signal (gRNA) was cloned at the multiple cloning site (MCS) between the *white* gene and an attB-docking site inside a plasmid used for *D*. *melanogaster* transformation^63^. For the first plasmid in this series, *U6-3-gRNA*^*βTub*^, *Drosophila U6-3* promoter was amplified from *Drosophila* genomic DNA with U6-1F and U6-2R primers, while the complete gRNA was PCR-assembled from two Ultramer^®^ gRNA-3F and gRNA-4R oligos synthesized by Integrated DNA Technology (IDT). To improve the efficiency of termination of gRNA transcription, a termination signal with 11 thymines was used in our design. In the successive plasmids, the *U6-3* promoter and gRNA’s scaffold was amplified from the *U6-3-gRNA*^*βTub*^ plasmid using the overlapping middle oligos designed to replace 20 bases that constitute a gRNA target (U6-1AF, U6-2A/B/CR, gRNA-3A/B/CF, and gRNA-4AR), and replaced by digesting the same plasmid at AscI and NotI sites. To assemble the set of plasmids with double gRNAs (*dsRNAs*), the *U6-3* promoter and *gRNA* was amplified as one fragment from the single gRNA (*sgRNA*) plasmids targeting female sex-determination genes with 2XgRNA-5F and 2XgRNA-6R primers, and cloned inside the *U6-3-gRNA*^*βTub*^ plasmid that was linearized at a BamHI site between the *white* gene and the U6-3 promoter. Each dgRNA plasmid had the same *gRNA*^*βTub*^ targeting *βTub85D* and a different *gRNA* targeting *Sxl*, *tra*, or *dsxF* expressed independently in the same direction (Supplementary Fig. 1). *Drosophila Cas9* plasmids and *gRNA* plasmids generated for this study were deposited at Addgene (Supplementary Fig. 1). To build the *βTub85D-GFP* construct, a 481-bp fragment directly upstream of *βTub* coding sequence was PCR amplified from *Drosophila* genomic DNA with βTub-F and βTub-R primers and cloned upstream of *GFP* into the *white* attB-docking site plasmid described above.

### Fly genetics and imaging

Flies were maintained under standard conditions at 25 °C. Embryo injections were carried out at Rainbow Transgenic Flies, Inc. (http://www.rainbowgene.com). The *Cas9* and *gRNA* constructs were inserted at the PBac{y + -attP-3B}KV00033 on the 3rd chromosome (Bloomington #9750) and the P{CaryP}attP1 on the 2nd chromosome (Bloomington #8621), respectively; while *βTub-GFP* construct was inserted at the M{3XP3-RFP.attP’}ZH-86Fa on the 3rd chromosome (Bloomington #24486). Transgenic flies were balanced with w^1118^; CyO/sna^Sco^ and w^1118^; TM3, Sb^1^/TM6B, and Tb^1^; and double balanced with w^1118^; CyO/Sp; Dr^1^/TM6C,Sb,Tb^1^. The *βTub*-*GFP* (on the 3rd chromosome) was double balanced and introgressed with *gRNA*^*βTub,Sxl*^, *gRNA*^*βTub,Tra*^, and *gRNA*^*βTub,DsxF*^, each on the 2nd chromosome, to generate trans-heterozygous balanced stocks (*dgRNA*/CyO; *βTub-GFP/*TM6C,Sb,Tb).

To test the efficiency of knockouts and corresponding phenotypes caused by *sgRNAs*, seven flies of each gender were crossed to generate trans-heterozygous F_1_ *sgRNA/+*; *nos-Cas9/+* flies for each combination of sgRNA; and their external morphology and fertility were examined. Both transgenes were identified on a fluorescent stereomicroscope with w+ eyes (s*gRNA*, *dgRNA*) and dsRed (*Cas9*). The sgRNA lines that caused knockout phenotypes were further tested as homozygous stocks with *nos-Cas9* flies in both directions using 10♂ and 10♀ flies for each replicate cross. DgRNA lines were tested bidirectionally with homozygous *nos-Cas9*, *vas-Cas9*, and *Ubi-Cas9* lines. In addition, *sgRNA*, *dgRNA*, and *Cas9* homozygous lines were crossed to w− flies in both directions to provide the comparison control. To test for the non-Mendelian dominant maternal effect of Cas9 loaded as a protein into embryos^38^, homozygous *dgRNA* flies were crossed to heterozygous *Cas9* flies; and phenotypes of *dgRNA/+;+/TM3*, *Sb* progeny with either maternal *Cas9* or paternal *Cas9* were compared. The F_1_ progeny from crosses with the paternal *Cas9* served as a control group to examine the dominant maternal effect of Cas9. To test the fertility of generated knockout flies with and without the *Cas9* gene, batches of 10–20 F_1_ males and females, or intersexes, were crossed to 15–20 female virgin and male flies, correspondingly, from w− and/or Cantos S stocks. Three or 4 days after the cross, the flies were passaged into fresh vials, and in a week, both vials were examined for the presence of any viable progeny. The fertility of an entire batch was scored as 100% when viable larvae were identified in a vial, or 0% when no progeny hatched in both vials. The vials containing intersexes and *wt* males were also examined for the presence of laid eggs. All crosses were repeated at the minimum three times to generate means and standard deviations for statistical comparisons and thus measure consistency and robustness of the results.

Flies were scored, examined, and imaged on the Leica M165FC fluorescent stereomicroscope equipped with the Leica DMC2900 camera. To generate images of adult flies, image stacks collected at different focal plates were compiled into single images in Helios Focus 6, and then edited in Adobe Photoshop CS6. To study internal anatomical features of intersex flies and sterile males, their reproductive organs were dissected in PBS buffer, examined, and imaged. To estimate the variation of knockout phenotypes, around 10–20 flies were dissected for each tested genotype.

### Developmental stage of *Sxl* lethality

To identify the developmental stage at which *Sxl* knockout females die, egg hatching and larval death rates were quantified for the *dgRNA*^*βTub,Sxl*^*/+*; *nos-Cas9*/+ trans-heterozygous flies. To quantify the egg hatching rate, three replicate crosses, each with 20–30 homozygous *nos-Cas9* female virgins and 10–20 *dgRNA*^*βTub,Sxl*^ males, were set up in embryo collection cages (Genesee Scientific 59–100) with grape juice agar plates. Three embryo collection cages with w− flies served as a comparison control. Batches of around 200 laid eggs were counted from each collection cage and followed for over 36 h to count the number of unhatched eggs. To quantify the rate of larval death, two batches of 50 emerged larvae were transferred from each agar plate to separate fly vials with food and raised to adults, and then the number and sex of emerged adults were recorded. To quantify the lethality at a pupal stage, a number of dead pupae were also recorded for each vial.

### RT-PCR of female- and male-specific transcripts of *Dsx*

To assess the effect of *tra* knockout on *dsx* splicing, we screened for female- and male-specific mRNA of *dsx* in *tra* knockout intersexes. Total RNA was extracted from adult w− male, w− female, and *tra* knockout (*dgRNA*^*βTub,Tra*^*/+*; *nos-Cas9*/+) intersex flies following the standard protocol of the MirVana miRNA isolation kit (Ambion). To remove DNA contamination, 2 µg was treated with TURBO^TM^ DNase using the TURBO DNA-free^TM^ Kit (Ambion). *Dsx* female and male splice variants were amplified with the SuperScript^®^ III One-Step RT-PCR Kit (Invitrogen) following the protocol. The same forward primer, Dsx-RT-1F, and two different reverse primers, DsxF-RT-2R and DsxM-RT-3R (Supplementary Table 11), were used to amplify either female or male transcripts, respectively. In total, 10 µL of PCR products were run on a 1% agarose gel to test PCR specificity, and the remaining 40 µL were purified using a QIAquick PCR purification kit (QIAGEN) or, when double bands were identified on a gel, gel-purified with a Zymoclean™ Gel DNA Recovery Kit (Zymo Research), then clean amplicons were sequenced in both directions using Sanger method at Source BioScience (https://www.sourcebioscience.com).

### Genotyping loci targeted with gRNAs

To examine the molecular changes that caused female lethality or masculinization and male sterility in the flies carrying *Cas9* and *gRNAs*, four genomic loci that include targets sites for four functional *gRNAs* (Supplementary Fig. 1) were amplified and sequenced. Single-fly genomic DNA preps were prepared by homogenizing a fly in 30 µl of a freshly prepared squishing buffer (10 mM Tris-Cl pH 8.0, 1 mM EDTA, 25 mM NaCl, and 200 μg/mL Proteinase K), incubating at 37 °C for 35 min, and heating at 95 °C for 2 min. In total, 2 µl of genomic DNA was used as a template in a 40-µL PCR reaction with LongAmp^®^ Taq DNA polymerase (NEB). The following primers (Supplementary Table 11) were used to amplify the loci with the corresponding gRNA targets: βTub-1AF and βTub-2AR for *βTubulin 85D*; Sxl-3BF and Sxl-4AR for *Sex lethal*; Tra-5F and Tra-6R for *Transformer*; Dsx-7F and Dsx-8R for *Double sex*. PCR products were purified using a QIAquick PCR purification kit (QIAGEN), and sequenced in both directions with Sanger method at Source BioScience. To characterize molecular changes at the targeted sites, sequence AB1 files were aligned against the corresponding reference sequences in SnapGene^®^ 4 and/or Sequencher™ 5.

### Competition assay of sterile males

To evaluate the competitiveness of the *βTub* knockout (*gRNA*^*βTub,Sxl*^*/+; nos-Cas9/+*) males, their ability to secure matings with females in the presence of *wt* males was evaluated. The w− males share the same genetic background with the *βTub* knockout males, and provide an ideal comparison. Two *wt*, one *wt*, one *wt* plus one *βTub* knockout, or two *βTub* knockout males were placed into a fly vial with ten w− virgins isolated on yeast paste for 2 days and allowed to court and mate with the females overnight (12 h) in the dark. To increase the male courtship drive, freshly emerged *dgRNA*^*βTub,Sxl*^*/+; nos-Cas9/+* and *wt* males were isolated from females and aged for 4 days before the competition assay. *Drosophila* females mate with multiple males during a lifespan; and in the absence of sperm transferred to spermatheca after copulation, female abstinence lasts for 1 day post copulation^64^. Therefore, after 12 h of mating, all males were removed from the vials, while the females were transferred into small embryo collection cages (Genesee Scientific 59–100) with grape juice agar plates. Three batches of eggs were collected within 36 h and unhatched eggs were counted. The decrease in fertility, estimated by a number of unhatched eggs, indicated the ability of a *gRNA*^*βTub,Sxl*^*/+; nos-Cas9/+* male to score successful matings with females in the presence of a *wt* male; and thus provided a readout of the competitiveness of *βTub* knockout males. A single *wt* male was used to test its ability to inseminate each of ten females in 12 h, and thus discriminate between a true competition or a dilution effect of two *wt* males.

### Survival curves to estimate longevity of pgSIT males

To compare differences in survival between pgSIT (*gRNA*^*βTub,Sxl*^*/+; nos-Cas9/+*) and *wt* males, average longevities for three experimental groups of males were estimated. We treated two types of pgSIT, one carrying paternal Cas9 and the other maternal Cas9, as separate experimental groups. Five replicates per each of three groups were applied to estimate survival curves. Males of each type were collected daily and aged in batches of 20 males per vial. Each replicate had from 40 to 75 males kept in two or four vials, respectively. Numbers of died flies were recorded every third day during the transfer of flies into a new vial with fresh food. We analyzed the interval-censored time-to-event (i.e., death) data for the three experimental groups by computing nonparametric maximum likelihood estimates (NPMLE) of the survival curves for each group, implemented in the R package interval^65^. The estimation procedure takes into account uncertainty introduced by the 3-day observation period. Bootstrap with 10,000 repetitions was applied to quantify median survival time and standard deviation.

### Mathematical modeling

To model the expected performance of pgSIT at suppressing local *Ae*. *aegypti* populations in comparison with currently available self-limiting suppression technologies—RIDL, fsRIDL, and IIT—we simulated release schemes for each using the MGDrivE simulation framework^43^ (https://marshalllab.github.io/MGDrivE/). This framework models the egg, larval, pupal, and adult mosquito life stages (both male and female adults are modeled) implementing a daily time step, overlapping generations and a mating structure in which adult males mate throughout their lifetime, while adult females mate once upon emergence, retaining the genetic material of the adult male with whom they mate for the duration of their adult lifespan. Density-independent mortality rates for the juvenile life stages are assumed to be identical and are chosen for consistency with the population growth rate in the absence of density-dependent mortality. Additional density-dependent mortality occurs at the larval stage, the form of which is taken from Deredec et al.^66^. The inheritance patterns for the pgSIT, RIDL, fsRIDL, and IIT systems are modeled within the inheritance module of the MGDrivE framework^43^, along with their impacts on adult lifespan, male mating competitiveness, and pupatory success. We implement the stochastic version of the MGDrivE framework to capture the random effects at low population sizes and the potential for population elimination. We simulated weekly releases over a period of 6 months into a randomly mixing population consisting of 10,000 adult females at equilibrium, with *Ae*. *aegypti* life history and intervention parameter values listed in Supplementary Table 10.

### Statistical analysis

Statistical analysis was performed in JMP 8.0.2 by SAS Institute Inc. Three to five biological replicates were used to generate statistical means for comparisons. *P* values were calculated for a two-sample Student’s *t* test with unequal variance. To test for significance of male sterilization, Pearson’s chi-squared tests for contingency tables were used to calculate *P* values. To test for differences between the inferred survival curves, we used Sun’s generalization of the log-rank test^67^. In addition, we performed pairwise post hoc tests of differences between the two pgSIT groups with conservative Bonferroni correction.

### Reporting Summary

Further information on experimental design is available in the Nature Research Reporting Summary linked to this article.

## Supplementary Information

Supplementary Information

Reporting Summary

## Data Availability

The data supporting the findings of this study are available within the paper and its Supplementary Information files. Complete annotated plasmid sequences and plasmid DNA are publicly available for order at Addgene. Transgenic flies have been made available for order from Bloomington *Drosophila* stock center. Accession codes for deposited plasmids and *Drosophila* stocks are listed in Supplementary Fig. 1.
