# Supplementary material for: Transforming insect population control with precision guided sterile males with demonstration in flies
Source: Nat Commun. 2019 Jan 8;10:84. doi: 10.1038/s41467-018-07964-7 (PMC6325135; doi:10.1038/s41467-018-07964-7)
Supplement: Supplementary file 1 — Supplementary Information [file 41467_2018_7964_MOESM1_ESM.pdf]

Supplementary Information

Transforming Insect Population Control with Precision Guided Sterile Males

Authors Affiliation

Nikolay P. Kandul[1], Junru Liu[1], Hector M. Sanchez C.[2], Sean L. Wu[2], John M. Marshall[2] and Omar S. Akbari[1,3†]

[1] Division of Biological Sciences, Section of Cell and Developmental Biology, University of California, San Diego, La Jolla, California, United States of America
[2] Division of Biostatistics and Epidemiology, School of Public Health, University of California, Berkeley, California, United States of America
[3] Tata Institute for Genetics and Society, University of California, San Diego, La Jolla, California, United States of America

Supplementary Information Contents

Supplementary Figures

Supplementary Fig. 1 Genetic constructs and corresponding transgenic lines developed in the study.

Supplementary Fig. 2 Characterization of three new homozygous lines expressing SpCas9.

Supplementary Fig. 3 Determination of developmental stage of female lethality.

Supplementary Fig. 4 Both male and female splice variants of *Dsx* are expressed in *βTub, Tra* knockout intersexes.

Supplementary Fig. 5 Biallelic mosaicism detected in the *βTub, sxl* knockout flies.

Supplementary Fig. 6 Biallelic mosaicism detected in the *βTub, tra* and *βTub, dsxF* knockout flies.

Supplementary Fig. 7 Sensitivity analysis for pgSIT system in *Aedes aegypti.*

Supplementary Fig. 8 Two novel gene drive designs based on lethal biallelic mosaicism.

Supplementary Fig. 9 One factory (blue) producing pgSIT eggs for distribution and release at remote locations (pink) worldwide.

Supplementary Tables

Supplementary Table 1 The F1 progeny from the crosses between homozygous single *gRNA* (*sgRNA/sgRNA*) and homozygous *nos-Cas9* (*nos-Cas9/nos-Cas9*).

Supplementary Table 2 Genotyping genomic loci targeted by gRNAs: (insertions/deletions) indels were found in trans-heterozygous flies.

Supplementary Table 3 The F1 progeny from the crosses between homozygous double gRNA (*sgRNA/dgRNA*) and homozygous Cas9 (*Cas9/Cas9*) lines.

Supplementary Table 4 Hatching rate of $gRNA^{\beta Tub.Sxl}$*/+; nos-Cas9/+* embryos.

Supplementary Table 5 Female pupal lethality in $dgRNA^{\beta Tub,Sxl}$*/+; nos-Cas9/+* embryos.

Supplementary Table 6 Phenotypic characteristics of trans-heterozygous flies carrying *Cas9* and double gRNAs (*dsRNA*).

Supplementary Table 7 The F1 progeny from the crosses between homozygous double gRNA (*dgRNA/dgRNA*) and heterozygous *Cas9* (*Cas9*/TM3, Sb).

Supplementary Table 8 Competitiveness of $dgRNA^{\beta Tub,Sxl}$*/+; nos-Cas9/+* males.

Supplementary Table 9 Longevity of $dgRNA^{\beta Tub,Sxl}$*/+; nos-Cas9/+* males.

Supplementary Table 10 Parameters used in *Aedes aegypti* population suppression model.

Supplementary Table 11 Primer sequences used in this study.

Supplementary References

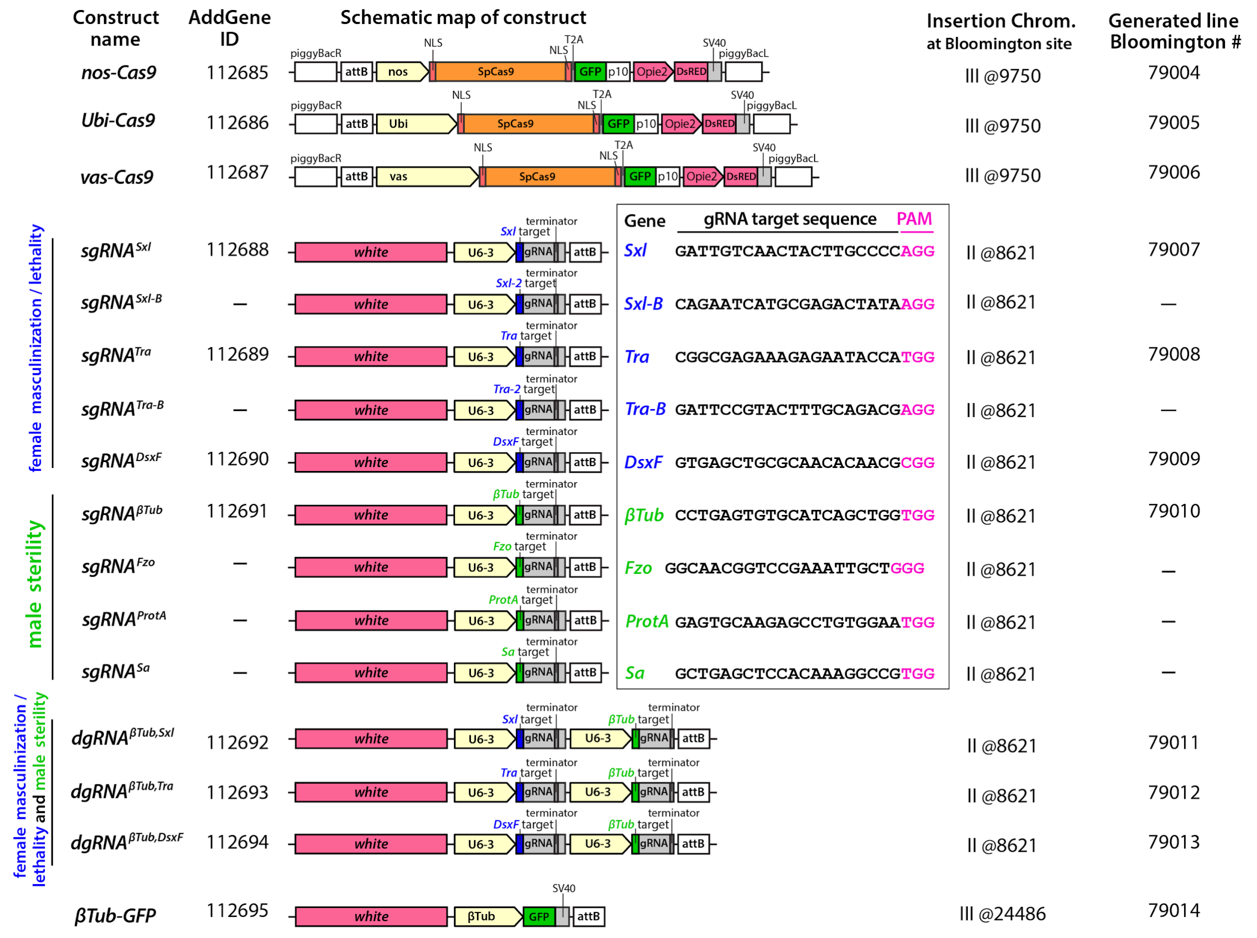

Supplementary Fig. 1 Genetic constructs and corresponding transgenic lines developed in the study. Schematics of all constructs engineered in this study, with functional constructs and flies deposited to Addgene.org and Bloomington Drosophila Stock Center, respectively. Gene names and gRNA target site sequences are presented in the box. The coding sequence of a *SpCas9* was flanked by two nuclear localization signals (NLS) at both ends and a self-cleaving T2A peptide with eGFP coding sequence at the C- end, serving as a visual indicator of Cas9 expression.

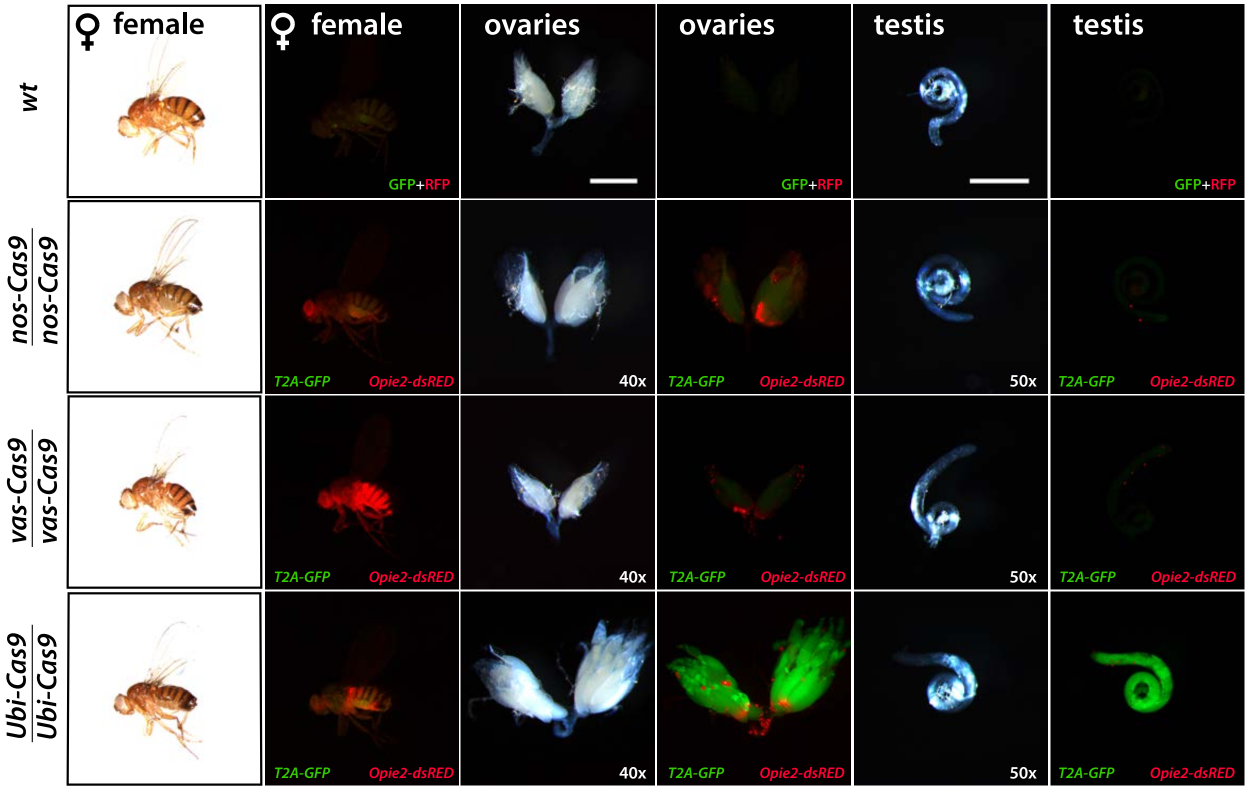

Supplementary Fig. 2 Characterization of three new homozygous lines expressing SpCas9. Three *Drosophila* lines supporting expression of *SpCas9* in strictly germline or germline together with somatic cells were developed. *Nanos-Cas9* (*nos-Cas9*), *vasa-Cas9* (*vas-Cas9*), and *Ubiquitin-63E* (*Ubi-Cas9*) were inserted at the same site on the 3rd chromosome using φC31-mediated integration. *Opie2-dsRed* trasgene served as a transgenesis marker and a self-cleaving T2A-eGFP sequence, which was attached to the 3'-end of *SpCas9* coding sequence, provided an indicator of Cas9 expression (Supplementary Fig. 1). Expression levels of dsRed and eGFP in each Cas9 line were compared to *wild type* (*wt*) flies. The Cas9-T2A-eGFP expression was mostly limited to female germline in *nos-Cas9* and *vas-Cas9* with a strong expression in *nos-Cas9*. *Ubi-Cas9* supported the strongest expression of Cas9, measured by eGFP, in both female and male germline, and in soma. Scale bars correspond to 500 μm.

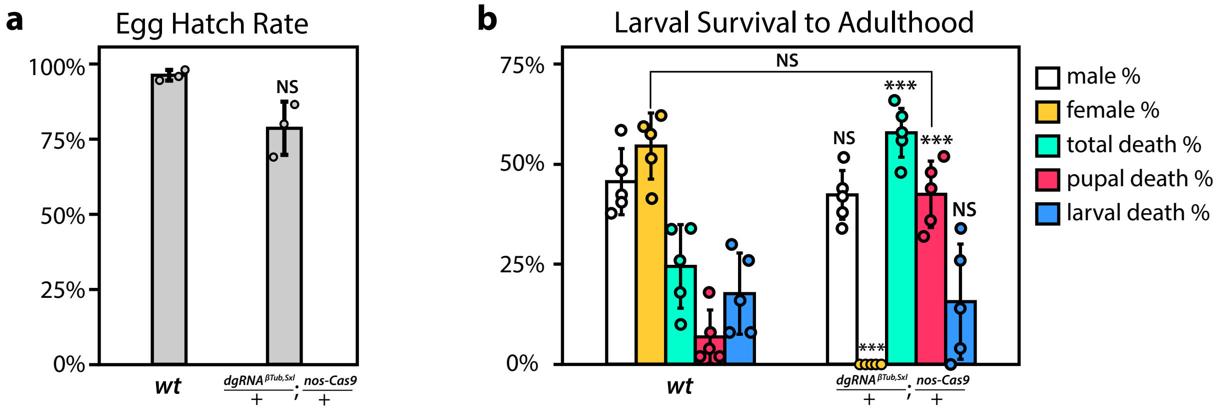

Supplementary Fig. 3 Determination of developmental stage of female lethality. **a** The percentage hatching rate estimated for *dgRNA*$^{\beta Tub,Sxl}$/+; *nos-Cas9*/+ eggs generated by crossing homozygous *nos-Cas9/nos-Cas9* ♀ and *dgRNA*$^{\beta Tub,Sxl}$/*dgRNA*$^{\beta Tub,Sxl}$ ♂ was not statistically different from that of the wild type (*wt*) eggs (Supplementary Table 5). **b** Rates of different outcomes for hatched *dgRNA*$^{\beta Tub,Sxl}$/+; *nos-Cas9*/+ larvae. Batches of 50 hatched larvae were raised to adults, their gender or developmental time of death was recorded (Supplementary Table 6). The majority of additional larval deaths happened during a pupal transition, and the percentage of pupal death was not statistically different from the *wt* ♀ percentage. Bar graphs show means ± one standard deviation. Statistical significance was calculated with a *t* test assuming unequal variance. (P <0.05^{NS}, P > 0.001***).

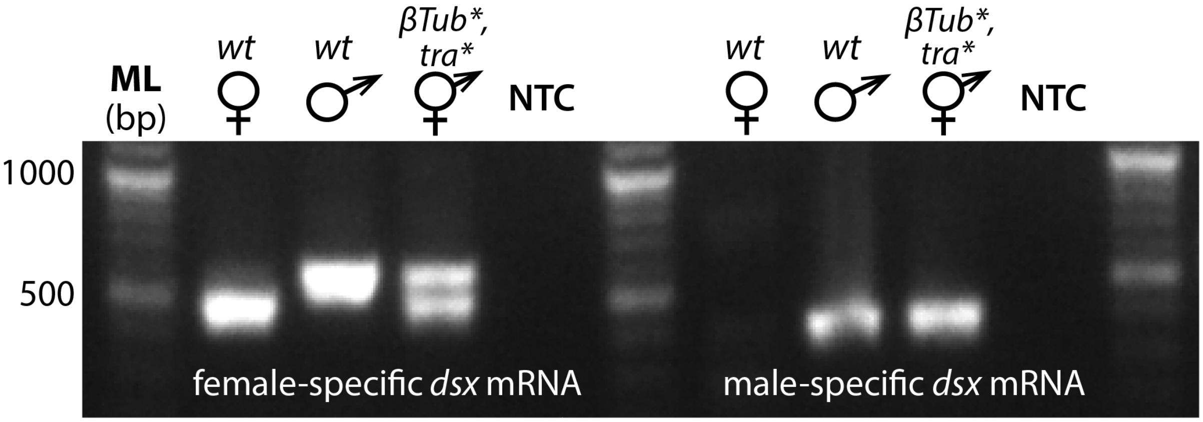

Supplementary Fig. 4 Both male and female splice variants of *Dsx* are expressed in *βTub*, *Tra* knockout intersexes. RT-PCR was used to assess female-specific and male-specific alternative splice variants of *dsx* comparing wild type (*wt*) females (♀), *wt* males (♂) and *dgRNA*$^{\beta Tub,Tra}$/+; *nos-Cas9*/+ intersexes (*βTub**, *Tra** ⚥). Both female and male-specific *dsx* transcripts were identified in *βTub**, *Tra** ⚥. Molecular ladder (ML) of double stranded DNA. No template control (NTC).

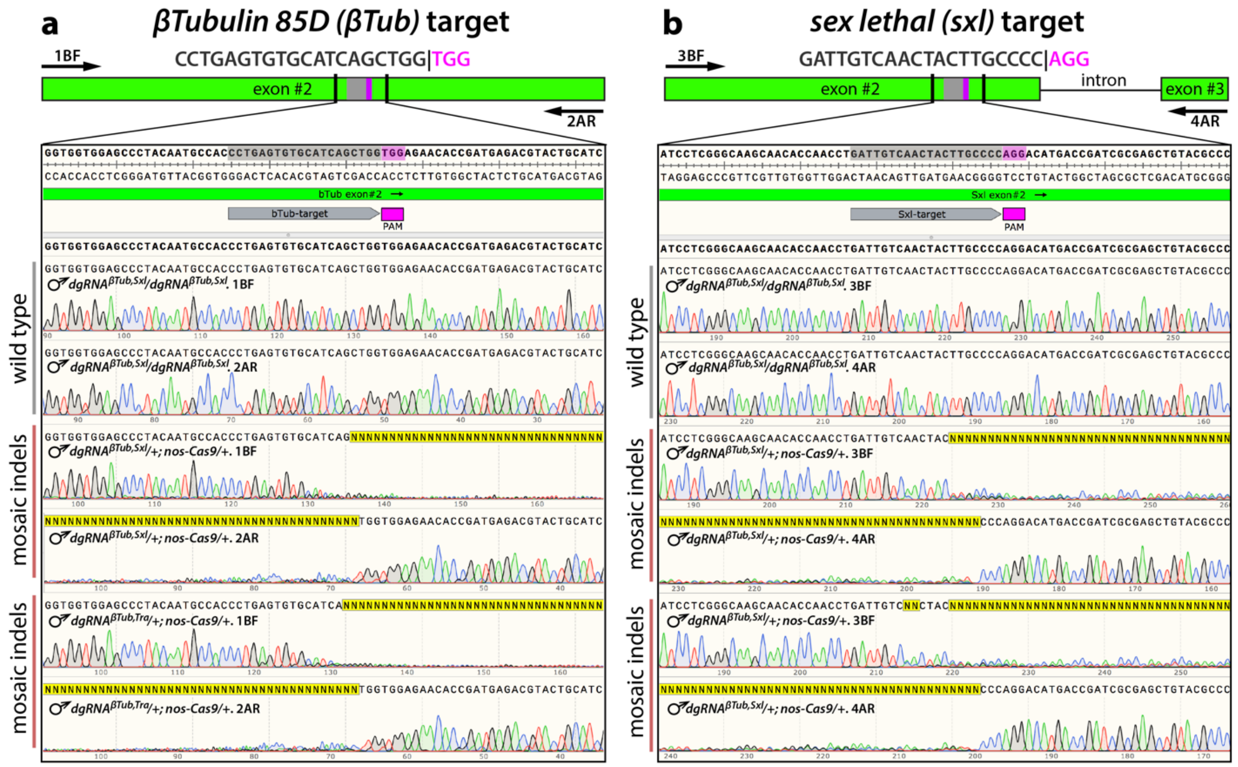

Supplementary Fig. 5 Biallelic mosaicism detected in the *βTub*, *sxl* knockout flies. **a** Trans-heterozygous *dgRNA*^{βTub,Sxl}/+; *nos-Cas9/+* sterile males (♂) had mosaic insertions / deletions (indels) precisely at the *βTub* target site. **b** Mosaic indels were also identified at the *Sxl* target site in the same *dgRNA*^{βTub,Sxl}/+; *nos-Cas9/+* sterile males (♂) and likely caused the pupal lethality of trans-heterozygous females (Fig. S3). Diagrams on the top present positions of gRNA target sites and primers used for PCR relative to genetic structures of targeted genes. Sequence reads from both ends inferred diversity of templates that specifically localized at the sites targeted with gRNAs in the sterile ♂, while the wild type ♂ had single alleles without any sequence ambiguity.

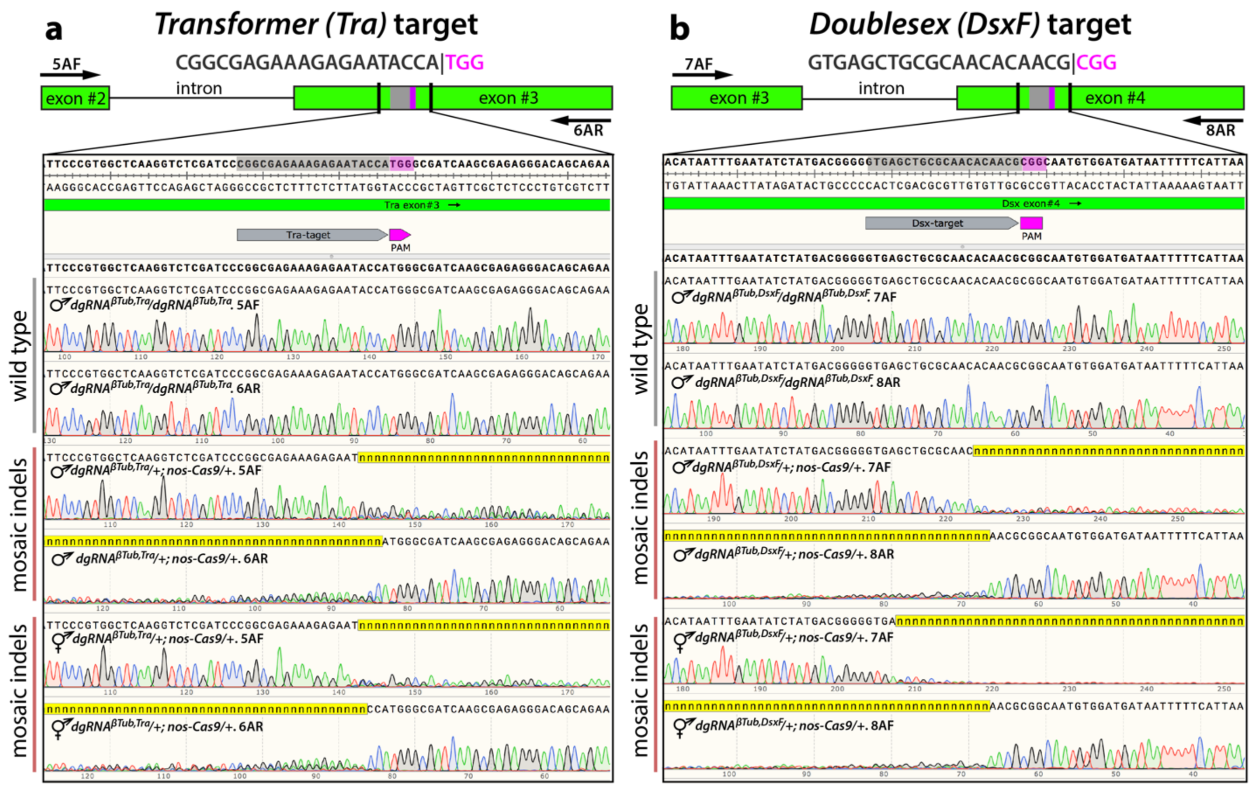

Supplementary Fig. 6 Biallelic mosaicism detected in the *βTub*, *tra* and *βTub*, *dsxF* knockout flies. **a** Trans-heterozygous $dgRNA^{\beta Tub,Tra}$*/+; nos-Cas9/+* sterile males (♂) and intersexes (⚥) had mosaic insertions / deletions (indels) located at the *Tra* site targeted by $dgRNA^{\beta Tub,Tra}$ double guide RNAs (dgRNA). **b** Mosaic indels were identified at the *DsxF* site targeted by the $dgRNA^{\beta Tub,DsxF}$ double gRNAs in $dgRNA^{\beta Tub,DsxF}$*/+; nos-Cas9/+* sterile ♂ and ⚥. Diagrams on the top show positions of gRNA targets and primers used for PCR relative to genetic structures of targeted genes. Sequence reads from both ends inferred diversity of templates that specifically localized at the sites targeted with gRNAs in sterile ♂ and ⚥, though the wild type ♂ had single alleles without any sequence ambiguity at both sites.

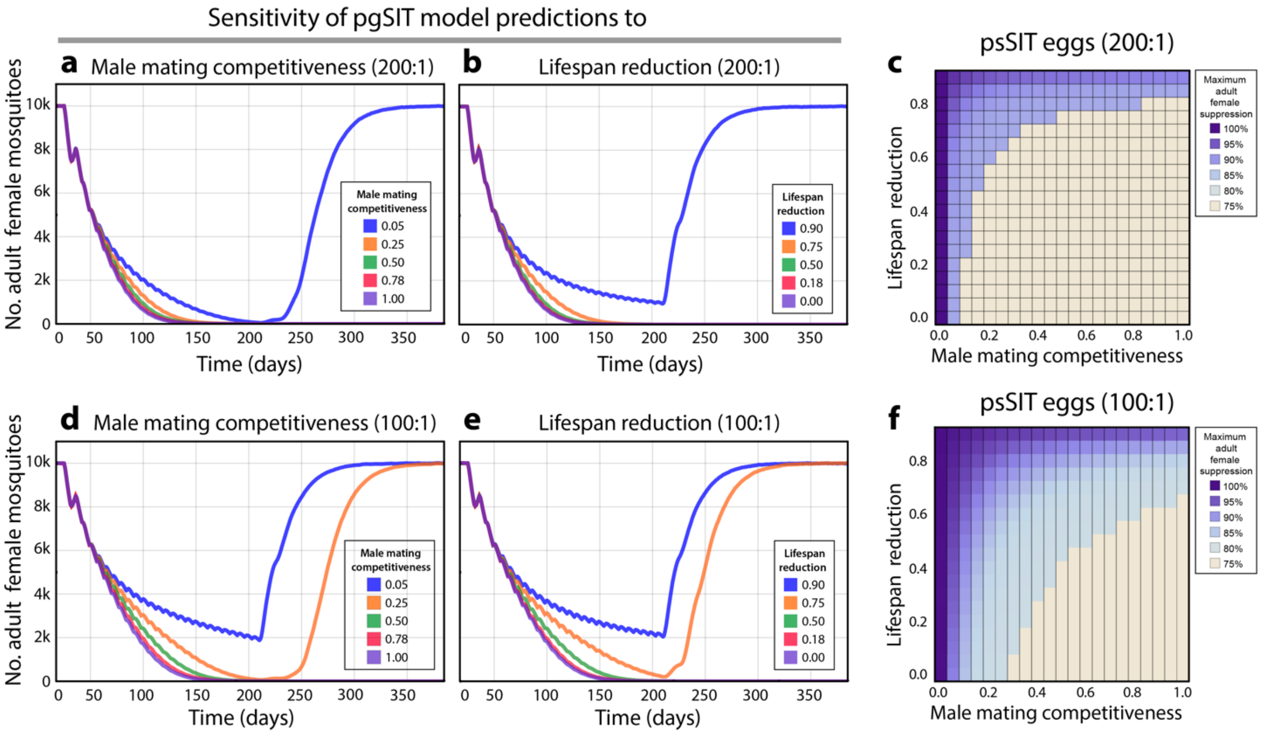

Supplementary Fig. 7 Sensitivity analysis for pgSIT system in *Aedes aegypti*. Sensitivity of pgSIT model predictions to male mating competitiveness, lifespan reduction and release ratio, keeping all other parameters constant as per Supplementary Table 10. Model predictions were computed using 250 realizations of the stochastic implementation of the MGDrivE simulation framework[1] for a randomly-mixing *Ae. aegypti* population of 10,000 adult females. **a** For a weekly release ratio of 200 eggs per wild adult and keeping lifespan reduction due to the pgSIT construct constant at 18%, elimination can be reliably achieved for a male mating competitiveness of 25%; but not for 5%, as is the case for RIDL adult males. **b** For a weekly release ratio of 200 eggs per wild adult and keeping male mating competitiveness constant at 78%, elimination can be reliably achieved for lifespan reductions less than or equal to 75%. **c** Varying lifespan reduction and male mating competitiveness simultaneously suggests a wide range of parameter values for which local *Ae. aegypti* elimination could be reliably achieved (tan tiles) given a weekly release ratio of 200 eggs per wild adult. **d** For a weekly release ratio of 100 eggs per wild adult and keeping lifespan reduction due to the pgSIT construct constant at 18%, elimination can be reliably achieved for a male mating competitiveness of 50%; but not for 25%. **e** For a weekly release ratio of 100 eggs per wild adult and keeping male mating competitiveness constant at 78%, elimination can be reliably achieved for lifespan reductions less than or equal to 50%. **f** Varying lifespan reduction and male mating competitiveness simultaneously suggests a smaller yet still wide range of parameter values for which local *Ae. aegypti* elimination could be reliably achieved (tan tiles) given a weekly release ratio of 100 eggs per wild adult.

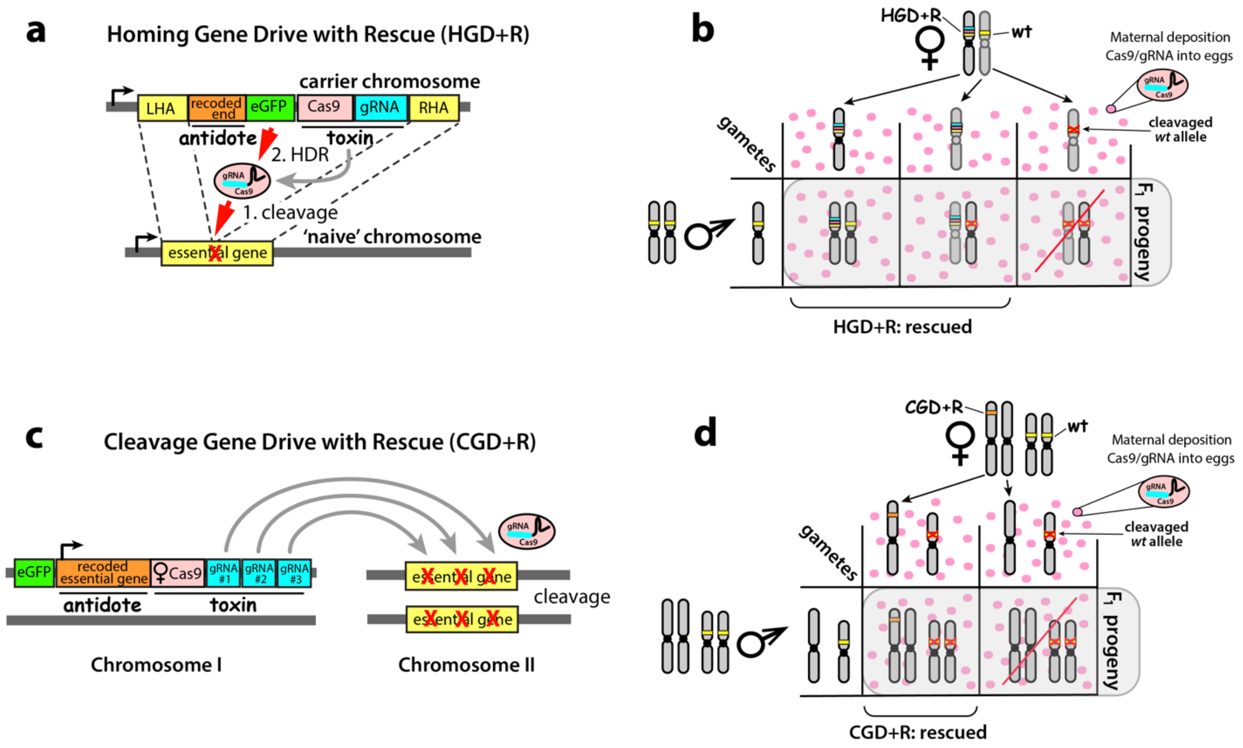

Supplementary Fig. 8 Two novel gene drive designs based on lethal biallelic mosaicism. **a** Schematic of **H**oming **G**ene **D**rive targeting an essential gene with a recoded **R**escue (HGD+R). The HGD+R expresses *Cas9* and a *gRNA* targeting an essential gene ('toxin'), a marker gene (eGFP), and the cleavage-resistant recorded portion of the essential gene that is being targeted by the gRNA/Cas9 complex ('antidote'), which can rescue the knockout phenotype, flanked by **L**eft and **R**ight **H**omology **A**rms (LHA and RHA). Mechanistically, once HGD+R is integrated precisely inside the targeted gene it will direct cleavage of the *wt* allele on the homologous 'naive' chromosome, and induce knockout mutations that will either result in lethal biallelic mosaicism, or convert the 'naive' chromosome into the carrier chromosome via homology directed repair (HDR). This ensures that only the progeny that inherit the HGD+R survive, while all progeny that inherit a cleaved allele perish due to non rescued lethal mosaicism. **b** The Punnett square depicts the genetics of how HGD+R achieves a 100% transmission rate in F1 progeny. Female heterozygous for HGD+R maternally deposits *Cas9*/*gRNA* complexes into every oocyte, and only zygotes that inherit the HDR+R would survive as F_1 progeny. Notably, HDR will convert *wt* alleles into HGD+R carriers and further increase numbers of surviving F_1 progeny and this non-Mendelian inheritance rate will depend on homing efficiencies. **c** Schematic of **C**leavage-only **G**ene **D**rive targeting essential gene with recoded **R**escue (CGD+R). The CGD+R expresses *Cas9* with multiple gRNAs targeting an essential gene in multiple locations ('toxin') to ensure robustness and stability of the drive, a marker gene (eGFP), and the cleavage-resistant recorded essential gene ('antidote') integrated at a separate genomic location from the target gene. Mechanistically, a CGD+R drive relies exclusively on cleavage with no HDR required for biased inheritance. **d** A Punnett square depicts the genetics of how CGD+R achieves 100% transmission rates in F1 progeny. The female heterozygous for CGD+R deposits *Cas9*/*gRNA* complexes into every oocyte, only the half of the zygotes that inherit the CDR+R in a mendelian fashion survive as F_1 progeny, while the other half that do not inherit CDR+R perish due to lethal biallelic mosaicism.

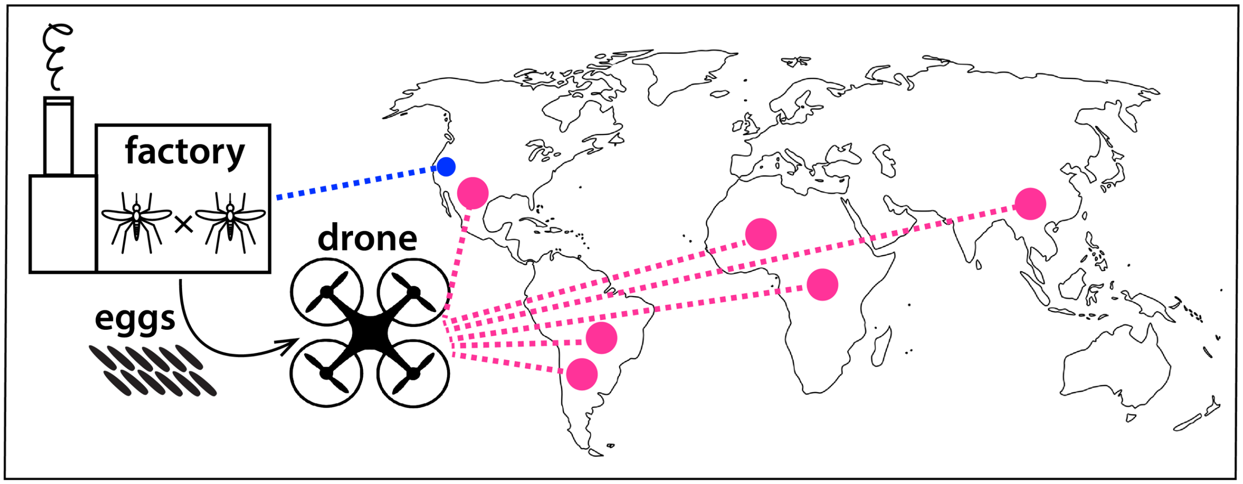

Supplementary Fig. 9 One factory (blue) producing pgSIT eggs for distribution and release at remote locations (pink) worldwide.

Supplementary Table 1. The F1 progeny from the crosses between homozygous single gRNA (sgRNA/sgRNA) and homozygous nos-Cas9 (nos-Cas9/nos-Cas9).

♀ Maternal Cas9															
	♂Wildtype (w-)			♂sgRNA[Slx]/sgRNA[Sxl]			♂sgRNA[Slx]/sgRNA[Tra]			♂sgRNA[DsxF]/sgRNA[DsxF]			♂sgRNA[bTub]/sgRNA[bTub]		
	♀	♂	⚥	♀	♂	⚥*	♀	♂	⚥*^	♀	♂	⚥*^	♀	♂*	⚥
**♀Wildtype (w-)**	-	-	-	119	118	0	69	57	0	66	63	0	80	77	0
**♀Wildtype (w-)**	-	-	-	57	56	0	80	64	0	67	61	0	92	101	0
**♀Wildtype (w-)**	-	-	-	86	79	0	72	61	0	72	75	0	88	94	0
**♀nos-Cas9/nos-Cas9**	84	83	0	0	67	0	0	56	53	0	64	61	67	65	0
**♀nos-Cas9/nos-Cas9**	70	72	0	0	65	0	0	60	54	0	56	53	61	58	0
**♀nos-Cas9/nos-Cas9**	52	49	0	0	61	0	0	57	55	0	54	54	63	67	0
**♀nos-Cas9/nos-Cas9**	-	-	-	0	88	0	0	87	68	0	113	53	115	76	0

♂ Paternal Cas9															
	♀ Wildtype (w-)			♀sgRNA[Slx]/sgRNA[Sxl]			♀sgRNA[Tra]/sgRNA[Tra]			♀sgRNA[DsxF]/sgRNA[DsxF]			♀sgRNA[bTub]/sgRNA[bTub]		
	♀	♂	⚥	♀	♂	⚥*	♀	♂	⚥*^	♀	♂	⚥*^	♀	♂*	⚥
**♂Wildtype (w-)**	-	-	-	92	88	0	62	57	0	65	57	0	59	53	0
**♂Wildtype (w-)**	-	-	-	72	67	0	77	72	0	63	60	0	66	62	0
**♂Wildtype (w-)**	-	-	-	76	69	0	81	80	0	73	70	0	73	71	0
**♂nos-Cas9/nos-Cas9**	74	70	0	0	87	0	0	63	67	0	91	88	87	75	0
**♂nos-Cas9/nos-Cas9**	70	67	0	0	90	0	0	72	70	0	97	100	95	86	0
**♂nos-Cas9/nos-Cas9**	87	74	0	0	82	0	0	81	79	0	95	87	95	90	0

Notes: At least ten females and ten males were set up for mating in each cross replicate, and their F1 progeny was scored and examined.

*100% steriliy

^ In some cases this is an underestimate due to difficulty to distinguish ⚥ from ♂

, each replicate cross of 7♀ x 10♂ parents, at the minimum.

Supplementary Table 2. Genotyping genomic loci targeted by gRNAs: (insertions/deletions) indels were found in transheterozygous flies.

#	Test	Fly line	Fly genotype	Fly sex	gRNA targets	Cas9 parent	*βTub* target	*Sxl* target	*Tra* target	*DsxF* target
1	wildtype	w-	w-	♀	–	–	wt	wt	wt	wt
2	wildtype	w-	w-	♀	–	–	wt	wt	wt	wt
3	wildtype	w-	w-	♂	–	–	wt	wt	wt	wt
4	wildtype	w-	w-	♂	–	–	wt	wt	wt	wt
5	wildtype	Canton S	w+	♀	–	–	wt	wt	wt	wt
6	wildtype	Canton S	w+	♀	–	–	wt	wt	wt	wt
7	wildtype	Canton S	w+	♂	–	–	wt	wt	wt	wt
8	wildtype	Canton S	w+	♂	–	–	wt	wt	wt	wt
9	control	*nos-Cas9*	nos-Cas9/CyO	♀	–	–	wt	wt	wt	wt
10	control	*nos-Cas9*	nos-Cas9/CyO	♀	–	–	wt	wt	wt	wt
11	control	*nos-Cas9*	nos-Cas9/nos-Cas9	♀	–	–	wt	wt	wt	wt
12	control	*nos-Cas9*	nos-Cas9/nos-Cas9	♀	–	–	wt	wt	wt	wt
13	control	*nos-Cas9*	nos-Cas9/CyO	♂	–	–	wt	wt	wt	wt
14	control	*nos-Cas9*	nos-Cas9/CyO	♂	–	–	wt	wt	wt	wt
15	control	*nos-Cas9*	nos-Cas9/nos-Cas9	♂	–	–	wt	wt	wt	wt
16	control	*nos-Cas9*	nos-Cas9/nos-Cas9	♂	–	–	wt	wt	wt	wt
17	control	*sgRNA*	sgRNA[βTub]/sgRNA[βTub]	♀	βTub	–	wt	–	–	–
18	control	*sgRNA*	sgRNA[βTub]/sgRNA[βTub]	♀	βTub	–	wt	–	–	–
19	control	*sgRNA*	sgRNA[βTub]/sgRNA[βTub]	♂	βTub	–	wt	–	–	–
20	control	*sgRNA*	sgRNA[βTub]/sgRNA[βTub]	♂	βTub	–	wt	–	–	–
21	control	*sgRNA*	sgRNA[Sxl]/sgRNA[Sxl]	♀	Sxl	–	–	wt	–	–
22	control	*sgRNA*	sgRNA[Sxl]/sgRNA[Sxl]	♀	Sxl	–	–	wt	–	–
23	control	*sgRNA*	sgRNA[Sxl]/sgRNA[Sxl]	♂	Sxl	–	–	wt	–	–
24	control	*sgRNA*	sgRNA[Sxl]/sgRNA[Sxl]	♂	Sxl	–	–	wt	–	–
25	control	*sgRNA*	sgRNA[Tra]/sgRNA[Tra]	♀	Tra	–	–	–	wt	–
26	control	*sgRNA*	sgRNA[Tra]/sgRNA[Tra]	♀	Tra	–	–	–	wt	–
27	control	*sgRNA*	sgRNA[Tra]/sgRNA[Tra]	♂	Tra	–	–	–	wt	–
28	control	*sgRNA*	sgRNA[Tra]/sgRNA[Tra]	♂	Tra	–	–	–	wt	–
29	control	*sgRNA*	sgRNA[DsxF]/sgRNA[DsxF]	♀	DsxF	–	–	–	–	wt
30	control	*sgRNA*	sgRNA[DsxF]/sgRNA[DsxF]	♀	DsxF	–	–	–	–	wt
31	control	*sgRNA*	sgRNA[DsxF]/sgRNA[DsxF]	♂	DsxF	–	–	–	–	wt
32	control	*sgRNA*	sgRNA[DsxF]/sgRNA[DsxF]	♂	DsxF	–	–	–	–	wt
33	control	*dgRNA*	dgRNA[βTub,Sxl]/dgRNA[βTub,Sxl]	♀	βTub & Sxl	–	wt	wt	–	–
34	control	*dgRNA*	dgRNA[βTub,Sxl]/CyO	♀	βTub & Sxl	–	wt	wt	–	–
35	control	*dgRNA*	dgRNA[βTub,Sxl]/dgRNA[βTub,Sxl]	♂	βTub & Sxl	–	wt	wt	–	–
36	control	*dgRNA*	dgRNA[βTub,Sxl]/CyO	♂	βTub & Sxl	–	wt	wt	–	–
37	control	*dgRNA*	dgRNA[βTub,Tra]/dgRNA[βTub,Tra]	♀	βTub & Tra	–	wt	–	wt	–
38	control	*dgRNA*	dgRNA[βTub,Tra]/CyO	♀	βTub & Tra	–	wt	–	wt	–
39	control	*dgRNA*	dgRNA[βTub,Tra]/dgRNA[βTub,Tra]	♂	βTub & Tra	–	wt	–	wt	–
40	control	*dgRNA*	dgRNA[βTub,Tra]/CyO	♂	βTub & Tra	–	wt	–	wt	–
41	control	*dgRNA*	dgRNA[βTub,DsxF]/dgRNA[βTub,DsxF]	♀	βTub & DsxF	–	wt	–	–	wt
42	control	*dgRNA*	dgRNA[βTub,DsxF]/CyO	♀	βTub & DsxF	–	wt	–	–	wt
43	control	*dgRNA*	dgRNA[βTub,DsxF]/dgRNA[βTub,DsxF]	♂	βTub & DsxF	–	wt	–	–	wt
44	control	*dgRNA*	dgRNA[βTub,DsxF]/CyO	♂	βTub & DsxF	–	wt	–	–	wt
45	experiment	*Cas9 & sgRNA*	sgRNA[βTub]/+; nos-Cas9/+	♀	βTub	♀	indels	–	–	–
46	experiment	*Cas9 & sgRNA*	sgRNA[βTub]/+; nos-Cas9/+	♀	βTub	♀	indels	–	–	–
47	experiment	*Cas9 & sgRNA*	sgRNA[βTub]/+; nos-Cas9/+	♂	βTub	♀	indels	–	–	–
48	experiment	*Cas9 & sgRNA*	sgRNA[βTub]/+; nos-Cas9/+	♂	βTub	♀	indels	–	–	–
49	experiment	*Cas9 & sgRNA*	sgRNA[Sxl]/+; nos-Cas9/+	♂	Sxl	♀	–	indels	–	–
50	experiment	*Cas9 & sgRNA*	sgRNA[Sxl]/+; nos-Cas9/+	♂	Sxl	♀	–	indels	–	–
51	experiment	*Cas9 & sgRNA*	sgRNA[Tra]/+; nos-Cas9/+	⚥	Tra	♀	–	–	indels	–
52	experiment	*Cas9 & sgRNA*	sgRNA[Tra]/+; nos-Cas9/+	⚥	Tra	♀	–	–	indels	–
53	experiment	*Cas9 & sgRNA*	sgRNA[Tra]/+; nos-Cas9/+	♂	Tra	♀	–	–	indels	–
54	experiment	*Cas9 & sgRNA*	sgRNA[Tra]/+; nos-Cas9/+	♂	Tra	♀	–	–	indels	–
55	experiment	*Cas9 & sgRNA*	sgRNA[DsxF]/+; nos-Cas9/+	⚥	DsxF	♀	–	–	–	indels
56	experiment	*Cas9 & sgRNA*	sgRNA[DsxF]/+; nos-Cas9/+	⚥	DsxF	♀	–	–	–	indels
57	experiment	*Cas9 & sgRNA*	sgRNA[DsxF]/+; nos-Cas9/+	♂	DsxF	♀	–	–	–	indels
58	experiment	*Cas9 & sgRNA*	sgRNA[DsxF]/+; nos-Cas9/+	♂	DsxF	♀	–	–	–	indels
59	experiment	Cas9 & gRNAs	dgRNA[βTub,Sxl]/+; nos-Cas9/+	♂	βTub & Sxl	♀	indels	indels	–	–
60	experiment	Cas9 & gRNAs	dgRNA[βTub,Sxl]/+; nos-Cas9/+	♂	βTub & Sxl	♀	indels	indels	–	–
61	experiment	Cas9 & gRNAs	dgRNA[βTub,Sxl]/+; nos-Cas9/+	♂	βTub & Sxl	♂	indels	indels	–	–

62	experiment	Cas9 & gRNAs	dgRNA[βTub,Sxl]/+; nos-Cas9/+	♂	βTub & Sxl	♂	indels	indels	–	–
63	experiment	*dgRNA*+loaded Cas9	dgRNA[βTub,Sxl]/+; TM3, Sb/+	♂	βTub & Sxl	♀	indels	indels	–	–
64	experiment	*dgRNA*+loaded Cas9	dgRNA[βTub,Sxl]/+; TM3, Sb/+	♂	βTub & Sxl	♀	indels	indels	–	–
65	experiment	*dgRNA*+loaded Cas9	dgRNA[βTub,Sxl]/+; TM3, Sb/+	♀	βTub & Sxl	♀	indels	indels	–	–
66	experiment	*dgRNA*+loaded Cas9	dgRNA[βTub,Sxl]/+; TM3, Sb/+	♀	βTub & Sxl	♀	indels	indels	–	–
67	experiment	*Cas9+loaded dgRNA*	CyO/+; nos-Cas9/+	♂	βTub & Sxl	♂	wt	wt	–	–
68	experiment	*Cas9+loaded dgRNA*	CyO/+; nos-Cas9/+	♂	βTub & Sxl	♂	wt	wt	–	–
69	experiment	*Cas9+loaded dgRNA*	CyO/+; nos-Cas9/+	♀	βTub & Sxl	♂	wt	wt	–	–
70	experiment	*Cas9+loaded dgRNA*	CyO/+; nos-Cas9/+	♀	βTub & Sxl	♂	wt	wt	–	–
71	experiment	*Cas9 & dgRNA*	dgRNA[βTub,Tra]/+; nos-Cas9/+	⚥	βTub & Tra	♀	indels	–	indels	–
72	experiment	*Cas9 & dgRNA*	dgRNA[βTub,Tra]/+; nos-Cas9/+	⚥	βTub & Tra	♀	indels	–	indels	–
73	experiment	*Cas9 & dgRNA*	dgRNA[βTub,Tra]/+; nos-Cas9/+	⚥	βTub & Tra	♂	indels	–	indels	–
74	experiment	*Cas9 & dgRNA*	dgRNA[βTub,Tra]/+; nos-Cas9/+	⚥	βTub & Tra	♂	indels	–	indels	–
75	experiment	*Cas9 & dgRNA*	dgRNA[βTub,Tra]/+; nos-Cas9/+	♂	βTub & Tra	♀	indels	–	indels	–
76	experiment	*Cas9 & dgRNA*	dgRNA[βTub,Tra]/+; nos-Cas9/+	♂	βTub & Tra	♀	indels	–	indels	–
77	experiment	*Cas9 & dgRNA*	dgRNA[βTub,Tra]/+; nos-Cas9/+	♂	βTub & Tra	♂	indels	–	indels	–
78	experiment	*Cas9 & dgRNA*	dgRNA[βTub,Tra]/+; nos-Cas9/+	♂	βTub & Tra	♂	indels	–	indels	–
79	experiment	*dgRNA*+loaded Cas9	dgRNA[βTub,Tra]/+; TM3, Sb/+	♂	βTub & Tra	♀	indels	–	indels	–
80	experiment	*dgRNA*+loaded Cas9	dgRNA[βTub,Tra]/+; TM3, Sb/+	♂	βTub & Tra	♀	indels	–	indels	–
81	experiment	*dgRNA*+loaded Cas9	dgRNA[βTub,Tra]/+; TM3, Sb/+	⚥	βTub & Tra	♀	indels	–	indels	–
82	experiment	*dgRNA*+loaded Cas9	dgRNA[βTub,Tra]/+; TM3, Sb/+	⚥	βTub & Tra	♀	indels	–	indels	–
83	experiment	*Cas9*+loaded dgRNA	CyO/+; nos-Cas9/+	♂	βTub & Tra	♂	wt	–	wt	–
84	experiment	*Cas9*+loaded dgRNA	CyO/+; nos-Cas9/+	♂	βTub & Tra	♂	wt	–	wt	–
85	experiment	*Cas9*+loaded dgRNA	CyO/+; nos-Cas9/+	♀	βTub & Tra	♂	wt	–	wt	–
86	experiment	*Cas9*+loaded dgRNA	CyO/+; nos-Cas9/+	♀	βTub & Tra	♂	wt	–	wt	–
87	experiment	*Cas9 & dgRNA*	dgRNA[βTub,DsxF]/+; nos-Cas9/+	⚥	βTub & DsxF	♀	indels	–	–	indels
88	experiment	*Cas9 & dgRNA*	dgRNA[βTub,DsxF]/+; nos-Cas9/+	⚥	βTub & DsxF	♀	indels	–	–	indels
89	experiment	*Cas9 & dgRNA*	dgRNA[βTub,DsxF]/+; nos-Cas9/+	⚥	βTub & DsxF	♂	indels	–	–	indels
90	experiment	*Cas9 & dgRNA*	dgRNA[βTub,DsxF]/+; nos-Cas9/+	⚥	βTub & DsxF	♂	indels	–	–	indels
91	experiment	*Cas9 & dgRNA*	dgRNA[βTub,DsxF]/+; nos-Cas9/+	♂	βTub & DsxF	♀	indels	–	–	indels
92	experiment	*Cas9 & dgRNA*	dgRNA[βTub,DsxF]/+; nos-Cas9/+	♂	βTub & DsxF	♀	indels	–	–	indels
93	experiment	*Cas9 & dgRNA*	dgRNA[βTub,DsxF]/+; nos-Cas9/+	♂	βTub & DsxF	♂	indels	–	–	indels
94	experiment	*Cas9 & dgRNA*	dgRNA[βTub,DsxF]/+; nos-Cas9/+	♂	βTub & DsxF	♂	indels	–	–	indels
95	experiment	*dgRNA*+loaded Cas9	dgRNA[βTub,DsxF]/+; TM3, Sb/+	♂	βTub & DsxF	♀	indels	–	–	indels
96	experiment	*dgRNA*+loaded Cas9	dgRNA[βTub,DsxF]/+; TM3, Sb/+	♂	βTub & DsxF	♀	indels	–	–	indels
97	experiment	*dgRNA*+loaded Cas9	dgRNA[βTub,DsxF]/+; TM3, Sb/+	⚥	βTub & DsxF	♀	indels	–	–	indels
98	experiment	*dgRNA*+loaded Cas9	dgRNA[βTub,DsxF]/+; TM3, Sb/+	⚥	βTub & DsxF	♀	indels	–	–	indels
99	experiment	*Cas9*+loaded dgRNA	CyO/+; nos-Cas9/+	♂	βTub & DsxF	♂	wt	–	–	wt
100	experiment	*Cas9*+loaded dgRNA	CyO/+; nos-Cas9/+	♂	βTub & DsxF	♂	wt	–	–	wt
101	experiment	*Cas9*+loaded dgRNA	CyO/+; nos-Cas9/+	♀	βTub & DsxF	♂	wt	–	–	wt
102	experiment	*Cas9*+loaded dgRNA	CyO/+; nos-Cas9/+	♀	βTub & DsxF	♂	wt	–	–	wt

Supplementary Table 3. The F1 progeny from the crosses between homozygous double gRNA (sgRNA/dgRNA) and homozygous Cas9 (Cas9/Cas9) lines.

♀ Maternal Cas9															
	♂Wildtype (w-)			♂dgRNA[bTub,Sxl]/sgRNA[bTub,Sxl]				♂dgRNA[bTub,Tra]/gRNA[bTub,Tra]				♂dgRNA[bTub,DsxF]/dgRNA[bTub,DsxF]			
	♀	♂	⚥	♀	♂	♂*	⚥*	♀	♂	♂*	⚥*^	♀	♂	♂ *	⚥*^
♀Wildtype (w-)	-	-	-	106	102	0	0	103	106	0	0	96	91	0	0
♀Wildtype (w-)	-	-	-	103	112	0	0	79	86	0	0	106	103	0	0
♀Wildtype (w-)	-	-	-	116	123	0	0	83	75	0	0	108	101	0	0
♀nos-Cas9/nos-Cas9	84	83	0	0	0	104	0	0	0	65	48	0	0	74	58
♀nos-Cas9/nos-Cas9	70	72	0	0	0	91	0	0	0	81	78	0	0	72	68
♀nos-Cas9/nos-Cas9	52	49	0	0	0	121	0	0	0	115	105	0	0	71	77
♀nos-Cas9/nos-Cas9	-	-	-	0	0	146	0	0	0	90	73	0	0	93	61
♀Ubi-Cas9/Ubi-Cas9	54	59	0	0	0	91	0	0	0	61	44	0	0	126	91
♀Ubi-Cas9/Ubi-Cas9	51	52	0	0	0	77	0	0	0	53	48	0	0	87	72
♀Ubi-Cas9/Ubi-Cas9	66	65	0	0	0	113	0	0	0	58	51	0	0	115	105
♀Ubi-Cas9/Ubi-Cas9	-	-	-	0	0	140	0	0	0	44	41	0	0	112	114
♀vas-Cas9/vas-Cas9	84	87	0	0	0	176	0	0	0	108	69	0	0	121	76
♀vas-Cas9/vas-Cas9	47	54	0	0	0	75	0	0	0	65	57	0	0	71	65
♀vas-Cas9/vas-Cas9	96	76	0	0	0	96	0	0	0	81	64	0	0	84	76
♀vas-Cas9/vas-Cas9	-	-	-	0	0	172	0	0	0	86	77	0	0	86	65

♂ Paternal Cas9															
	♀ Wildtype (w-)			♀dgRNA[bTub,Sxl]/dgRNA[bTub,Sxl]				♀dgRNA[bTub,Tra]/dgRNA[bTub,Tra]				♀dgRNA[bTub,DsxF]/dgRNA[bTub,DsxF]			
	♀	♂	⚥	♀	♂	♂*	⚥*^	♀	♂	♂*	⚥*^	♀	♂	♂ *	⚥*^
♂Wildtype (w-)	-	-	-	111	99	0	0	82	61	0	0	77	77	0	0
♂Wildtype (w-)	-	-	-	121	110	0	0	80	65	0	0	82	76	0	0
♂Wildtype (w-)	-	-	-	111	96	0	0	63	61	0	0	99	98	0	0
♂nos-Cas9/nos-Cas9	74	70	0	0	0	65	0	0	0	69	62	0	0	57	53
♂nos-Cas9/nos-Cas9	70	67	0	0	0	127	0	0	0	120	136	0	0	168	130
♂nos-Cas9/nos-Cas9	87	74	0	0	0	86	0	0	0	105	91	0	0	63	58
♂nos-Cas9/nos-Cas9	-	-	-	0	0	111	0	0	0	83	54	0	0	59	40
♂Ubi-Cas9/Ubi-Cas9	52	60	0	0	0	67	0	0	0	53	49	0	0	96	83
♂Ubi-Cas9/Ubi-Cas9	54	51	0	0	0	57	0	0	0	46	41	0	0	68	42

♂Ubi-Cas9/Ubi-Cas9	58	53	0	0	0	105	0	0	0	68	71	0	0	61	52
♂Ubi-Cas9/Ubi-Cas9	-	-	-	0	0	125	0	0	0	59	29	0	0	79	67
♂vas-Cas9/vas-Cas9	68	46	0	0	0	149	0	0	0	138	120	0	0	79	79
♂vas-Cas9/vas-Cas9	72	71	0	0	0	54	0	0	0	171	150	0	0	270	145
♂vas-Cas9/vas-Cas9	83	68	0	0	0	58	0	0	0	45	37	0	0	56	42
♂vas-Cas9/vas-Cas9	-	-	-	0	0	115	0	0	0	110	102	0	0	89	72

Notes: At least ten females and ten males were set up for mating in each cross replicate, and their F1 progeny was scored and examined.

***100% steriliy**

^ In some cases this is an underestimate due to difficulty to distinguish ⚥ from ♂

Supplementary Table 4. Hatching rate of gRNA[bTub.Sxl]/+; nos-Cas9/+ embryos.

Progeny genotype	laid eggs	hatched eggs	Hatch Rate
wt	201	190	94.52736318
wt	203	199	98.02955665
wt	216	207	95.83333333
dgRNA[bTub,Sxl]/+; nos-Cas9/+	200	173	86.5
dgRNA[bTub,Sxl]/+; nos-Cas9/+	200	160	80
dgRNA[bTub,Sxl]/+; nos-Cas9/+	200	138	69

Supplementary Table 5. Female pupal lethality in dgRNAbTub,Sxl/+; nos-Cas9/+ embryos.

**Line**	seed larvae #	♂emerged	♀emerged	♂%	♀%	TotalDeath%	PupalDeath	PupalDeath%	LarvalDeath%
dgRNA[bTub,Sxl]/+; nos-Cas9/+	50	26	0	52.00	0	48	24	48	0
dgRNA[bTub,Sxl]/+; nos-Cas9/+	50	22	0	44.00	0	56	26	52	4
dgRNA[bTub,Sxl]/+; nos-Cas9/+	50	21	0	42.00	0	58	22	44	14
dgRNA[bTub,Sxl]/+; nos-Cas9/+	50	17	0	34.00	0	66	16	32	34
dgRNA[bTub,Sxl]/+; nos-Cas9/+	50	19	0	38.00	0	62	18	36	26
w-	50	14	19	42.42	57.58	34	2	4	30
w-	50	15	22	40.54	59.46	26	9	18	8
w-	50	16	17	48.48	51.52	34	4	8	26
w-	50	24	17	58.54	41.46	18	1	2	16
w-	50	17	28	37.78	62.22	10	1	2	8

Supplementary Table 6. Phenotypic characteristics of trans-heterozygous flies carrying Cas9 and double gRNAs (dsRNA).

**Phenotype**	***dgRNA$^{\beta Tub, Sxl}$/+; Cas9/+***		***dgRNA$^{\beta Tub, Tra}$/+; Cas9/+***		***dgRNA$^{\beta Tub, DsxF}$/+; Cas9/+***	
	♂	♀	♂	♀	♂	♀
Viability	viable	100% lethal	viable	viable	viable	viable
External morphology	normal male	N/A	normal male	intersex	normal male	intersex
Fertility	100% sterile	N/A	100% sterile	100% sterile	100% sterile	100% sterile
Ovaries	absent	N/A	absent	rudimentary, variable	absent	developed or rudimentary
Egg produced	N/A	N/A	N/A	few, rare	N/A	numerous, frequent
Egg laid	N/A	N/A	N/A	never	N/A	never
Testis	developed	N/A	developed	absent	developed	absent
Male accessory glands	developed	N/A	developed	developed	developed	developed
Sex combs	developed	N/A	developed	developed, variable	developed	absent

Note: The phenotypic features other than lethality and sterility show variable manifestation independently of which Cas9 line (*nos-Cas9*, *Ubi-Cas9*, and *vas-Cas9*) was used or how Cas9 was inherited (maternal and paternal Cas9).

Supplementary Table 7. The F1 progeny from the crosses between homozygous double gRNA (dgRNA/dgRNA) and heterozygous Cas9 (Cas9/TM3, Sb).

♂Paternal Cas9																		
	♀dgRNA[bTub,Slx]/dgRNA[bTub,Slx]						♀dgRNA[bTub,Tra]/dgRNA[bTub,Tra]						♀dgRNA[bTub,DsxF]/dgRNA[bTub,DsxF]					
	♀	♀TM3	♂*	♂TM3	⚥*	⚥TM3*	♀	♀TM3	♂*	♂TM3	⚥*^	⚥TM3*	♀	♀TM3	♂*	♂TM3	⚥*^	⚥TM3*
♂nos-Cas9/TM3, Sb	0	49	52	49	0	0	0	46	56	54	51	0	0	40	44	39	44	0
♂nos-Cas9/TM3, Sb	0	53	61	54	0	0	0	47	57	46	54	0	0	46	56	42	54	0
♂nos-Cas9/TM3, Sb	0	57	63	59	0	0	0	52	57	45	60	0	0	60	42	53	42	0
♂Ubi-Cas9/TM3, Sb	0	48	50	37	0	0	0	68	56	72	61	0	0	47	36	40	24	0
♂Ubi-Cas9/TM3, Sb	0	46	51	62	0	0	0	60	39	58	37	0	0	65	73	50	45	0
♂Ubi-Cas9/TM3, Sb	0	56	47	39	0	0	0	58	38	56	38	0	0	45	51	37	31	0
♂vas-Cas9/TM3, Sb	0	47	51	44	0	0	0	37	48	35	42	0	0	41	57	45	52	0
♂vas-Cas9/TM3, Sb	0	45	46	43	0	0	0	42	46	42	44	0	0	45	45	42	48	0
♂vas-Cas9/TM3, Sb	0	44	49	41	0	0	0	55	37	46	58	0	0	57	44	54	54	0

♀ Maternal Cas9																		
	♂dgRNA[bTub,Slx]/dgRNA[bTub,Slx]						♂dgRNA[bTub,Tra]/dgRNA[bTub,Tra]						♂dgRNA[bTub,DsxF]/dgRNA[bTub,DsxF]					
	♀	♀TM3	♂*	♂TM3	⚥*	⚥TM3*	♀	♀TM3	♂*	♂TM3	⚥*^	⚥TM3*^	♀	♀TM3	♂*	♂TM3	⚥*^	⚥TM3*^
♀nos-Cas9/TM3, St	0	43	55	38	0	0	0	0	46	41	48	39	0	0	49	42	46	41
♀nos-Cas9/TM3, St	0	41	46	32	0	0	0	0	36	24	32	21	0	0	57	31	53	35
♀nos-Cas9/TM3, St	0	43	43	65	0	0	0	0	36	30	31	24	0	0	39	38	40	33
♀nos-Cas9/TM3, St	0	80	96	70	0	0	0	0	47	45	43	40	0	0	50	39	26	25
♀Ubi-Cas9/TM3, Sb	0	0	35	21	0	0	0	0	32	30	24	23	0	0	42	23	31	37
♀Ubi-Cas9/TM3, Sb	0	0	46	41	0	0	0	0	48	32	41	45	0	0	58	36	46	28
♀Ubi-Cas9/TM3, Sb	0	0	40	29	0	0	0	0	39	38	39	27	0	0	47	24	45	16
♀Ubi-Cas9/TM3, Sb	0	0	45	42	0	0	0	0	46	41	34	28	0	0	54	33	37	19
♀vas-Cas9/TM3, Sb	0	88	91	69	0	0	0	0	49	25	53	39	0	0	64	35	74	56
♀vas-Cas9/TM3, Sb	0	66	64	51	0	0	0	0	53	59	49	46	0	0	39	39	41	27
♀vas-Cas9/TM3, Sb	0	110	77	107	0	0	0	0	93	58	100	7	0	0	76	57	79	53
♀vas-Cas9/TM3, Sb	0	85	69	39	0	0	0	0	48	36	31	42	0	0	55	30	66	31

Notes: At least seven females and seven males were set up for mating in each cross replicate, and their F1 progeny was scored and examined.

*100% steriliy

^ In some cases this is an underestimate due to difficulty to distinguish ⚥ from ♂

Supplementary Table 8. Competitiveness of dgRNAbTub,Sxl/+; nos-Cas9/+ males.

Class # in Fig. 4	♂ genotype	laid eggs	unhatched eggs	hatched eggs	EggLaid %	hatching %
2	2 wt ♂	131	6	125	65.83	95.42
2	2 wt ♂	60	18	42	30.15	70.00
2	2 wt ♂	141	10	131	70.85	92.91
2	2 wt ♂	146	5	141	73.37	96.58
2	2 wt ♂	89	26	63	44.72	70.79
1	1 wt ♂	124	6	118	62.31	95.16
1	1 wt ♂	129	24	105	64.82	81.40
1	1 wt ♂	127	18	109	63.82	85.83
1	1 wt ♂	104	5	99	52.26	95.19
1	1 wt ♂	165	32	133	82.91	80.61
3	1 wt ♂ & 1 bTub*, sxl* ♂	144	50	94	72.36	65.28
3	1 wt ♂ & 1 bTub*, sxl* ♂	108	71	37	54.27	34.26
3	1 wt ♂ & 1 bTub*, sxl* ♂	85	56	29	42.71	34.12
3	1 wt ♂ & 1 bTub*, sxl* ♂	72	31	41	36.18	56.94
3	1 wt ♂ & 1 bTub*, sxl* ♂	139	71	68	69.85	48.92
4	2 bTub*, Sxl* ♂	145	145	0	72.86	0.00
4	2 bTub*, Sxl* ♂	169	169	0	84.92	0.00
4	2 bTub*, Sxl* ♂	97	97	0	48.74	0.00
4	2 bTub*, Sxl* ♂	199	199	0	100.00	0.00
4	2 bTub*, Sxl* ♂	101	101	0	50.75	0.00

Supplementary Table 9. Longevity of dgRNAbTub,Sxl/+; nos-Cas9/+ males.

replicate #	description of ♂ \ **day#**	**1**	**3**	**6**	**9**	**12**	**15**	**18**	**21**	**24**	**27**	**30**	**33**	**36**	**39**	**42**	**45**	**48**	**51**	**54**	**57**	**60**	**63**	**66**	**69**	**72**	**75**	**78**	**81**	**84**	**87**	**90**	**93**
1	♂, control w-	50	47	45	45	45	44	44	39	34	26	25	18	12	5	1	1	1	1	1	0	0	0	0	0	0	0	0	0	0	0	0	0
2	♂, control w-	50	43	39	38	38	37	32	32	30	25	24	16	13	7	1	1	1	1	1	1	1	1	1	1	1	1	1	0	0	0	0	0
3	♂, control w-	50	49	45	45	45	45	45	44	44	42	35	25	13	3	1	1	1	1	1	1	1	1	1	1	0	0	0	0	0	0	0	0
4	♂, control w-	75	62	62	60	58	58	58	57	55	54	51	49	42	23	19	18	15	15	8	7	4	2	2	2	0	0	0	0	0	0	0	0
5	♂, control w-	50	47	47	46	46	41	41	40	40	38	37	35	24	17	16	9	6	6	3	1	0	0	0	0	0	0	0	0	0	0	0	0
1	♂, dgRNA[bTub,Sxl]/+;nos-cas9(♂)/+	50	50	43	43	43	42	42	42	41	40	39	38	38	37	33	30	30	29	25	16	13	11	9	4	3	2	2	2	2	1	0	0
2	♂, dgRNA[bTub,Sxl]/+;nos-cas9(♂)/+	50	50	48	43	42	41	41	40	40	40	40	39	39	37	33	31	31	31	27	21	15	15	13	8	6	4	3	3	1	1	0	0
3	♂, dgRNA[bTub,Sxl]/+;nos-cas9(♂)/+	40	39	39	38	37	37	37	37	36	36	36	36	36	35	33	30	30	27	23	22	17	13	6	5	2	2	2	1	1	0	0	0
4	♂, dgRNA[bTub,Sxl]/+;nos-cas9(♂)/+	40	38	38	37	37	35	35	34	33	33	33	33	31	29	27	27	22	19	17	15	10	0	0	0	0	0	0	0	0	0	0	0
5	♂, dgRNA[bTub,Sxl]/+;nos-cas9(♂)/+	40	40	39	39	38	37	37	37	37	36	35	35	32	32	30	30	26	22	19	18	15	12	10	7	5	3	3	3	0	0	0	0
1	♂, dgRNA[bTub,Sxl]/+;nos-cas9(♀)/+	50	45	43	42	42	42	42	41	41	41	41	40	38	38	38	38	38	37	33	23	20	15	9	2	2	1	1	1	1	1	0	0
2	♂, dgRNA[bTub,Sxl]/+;nos-cas9(♀)/+	50	50	50	50	50	48	47	46	46	46	46	46	43	42	41	38	36	34	34	26	19	18	14	7	5	5	4	2	2	1	1	0
3	♂, dgRNA[bTub,Sxl]/+;nos-cas9(♀)/+	50	49	49	48	48	47	47	47	46	45	41	38	38	37	36	36	35	30	24	20	20	17	9	8	5	4	4	3	2	0	0	0
4	♂, dgRNA[bTub,Sxl]/+;nos-cas9(♀)/+	75	73	72	72	72	71	70	69	68	68	66	65	64	59	56	55	54	46	39	29	21	20	12	11	9	7	6	1	1	0	0	0
5	♂, dgRNA[bTub,Sxl]/+;nos-cas9(♀)/+	50	49	49	49	48	48	48	46	46	45	45	45	43	42	39	35	35	25	18	16	13	3	3	1	1	1	0	1	0	0	0	0

Supplementary Table 10. Parameters used in *Aedes aegypti* population suppression model.

Strategy	Parameter	Value	Reference
General	Egg production per female (day^{-1})	20	[2]
	Duration of egg stage (days)	5	[3]
	Duration of larval stage (days)	6	[3]
	Duration of pupal stage (days)	4	[3]
	Daily population growth rate (day^{-1})	1.175	[4]
	Daily mortality risk of adult stage (day^{-1})	0.090	[5–7]
	Adult female population size	10,000	[8]
IIT	Reduction in adult male lifespan	0.50	[9,10]
	Male mating competitiveness	1.00	[11,12]
RIDL / fsRIDL	Reduction in adult male lifespan	0.18	[13]
	Male mating competitiveness	0.05	[8,14]
pgSIT	Reduction in adult male lifespan	0.18	[13]
	Male mating competitiveness	0.78	Fig. 4B

Supplementary Table 11. Primer sequences used in this study.

Primer name	Primer sequence (5'–3')
nos-F	CTCGACGGTCACGGCGGGCATGTCGACGCAAAGCTTCGACCGTTTTAACCTCGAAATATG
nos-R	GTCTCCGTCGTGGTCCTTATAGTCCATctcgaGGCGAAAATCCGGGTCGAAAGTTACGG
ubi-F	TCGACGGTCACGGCGGGCATGTCGACGCGGCCGCCGCGCAGATCGCCGATGGGCGTGGCG
ubi-R	GTCTCCGTCGTGGTCCTTATAGTCCATATTTAAATTCTGCGGGTCAAAATAGAGATGTGGA
vas-F	CTCGACGGTCACGGCGGGCATGTCGACGCCTGCAGCTGGTTGTAGGTGCAGTTGCGCTTC
vas-R	GTAGTCTCCGTCGTGGTCCTTATAGTCCATctcgaATTGATATTTTTTTTTAATTTGGCCTGCCTTTC
U6-1F	ATTGGGAATTGGGCAATATTTAAATGGCGGCGCGCCGAATTCTTTTTTGCTCACCTGTGA
U6-2R	CTCTAAAACCCAGCTGATGCACACTCAGGCGACGTTAAATTGAAAATAGGTC
gRNA-3F	ATATATAGACCTATTTTCAATTTAACGTCG**CCTGAGTGTGCATCAGCTGG**GTTTTAGAGCTAGAAATAGCAAGTTAAAATAAGGCTAGTCC
gRNA-4R	CGTCGACACTAGTGGATCTCTAGAGGTACCAAAAAAAAAAGCACCGACTCGGTGCCACTTTTTCAAGTTGATAACGGACTAGCCTTATTTTAACTTGCTATTTCTAGCTCTAAAAC
U6-1AF	GCTAGCGGATCCGGGAATTGGGAATTGGGCAATATTTAAATGGC
U6-2(A-C)R	GCTATTTCTAGCTCTAAAAC**NNNNNNNNNNNNNNNNNNNN**CGACGTT
gRNA-3(A-C)F	CGTCG**NNNNNNNNNNNNNNNNNNNN**GTTTTAGAGCTAGAAATAGC
gRNA-4AR	GCGGCTTCGAGACCGTGACCTACATCGTCGAC
2XgRNA-5F	TGTATGCTATACGAAGTTATGCTAGCGGATCCGAATTCTTTTTTGCTCACCTGTGATTGC
2XgRNA-6R	ATTTAAATATTGCCCAATTCCCAATTCCCGGTACCAAAAAAAAAAGCACCGACTCGGTGC
βTub-F	GGAATTGGGAATTGGGCAATATTTAAATGGCGGTCGACCCATCCCATTATACACCCATAT
βTub-R	GTGAACAGCTCCTCGCCCTTGCTCACCATTTTGATAGTAAAGTTAGGGCCCCTTTTTCAC
Dsx-RT-1F	CGGGAACATCGGTGATCACTAGCGCCG
DsxF-RT-2R	CGCGCGATTTCACCGAAGCATTAAGCTAATATGTGATGCTG
DsxM-RT-3R	CGATGCTCGGGTAGGTCAGGTACATGGGC
βTub-1BF	GGCTTCCAGCTGACCCACTCGCTGGG
βTub-2AR	CCAGATGGTTCAGGTCACCGTAGGTGG
Sxl-3BF	CACGTTATGCGTTCTCGCCACAGGATACAG
Sxl-4AR	GCGAGTCCATTTCCGATGTGAAGTCCACG
Tra-5F	GGATGAGAATGAACGCTGGACGACCAGAC
Tra-6R	CTGTGATCTGGATCTGGAGCGAGTGCG
Dsx-7F	CCTTGGGAGCTGATGCCACTCATGTATGTG
Dsx-8R	CGCGCGATTTCACCGAAGCATTAAGCTAATATGTGATGCTG

Supplementary references

1. Sanchez C., H. M., Wu, S. L., Bennett, J. B. & Marshall, J. M. MGDrivE: A modular simulation framework for the spread of gene drives through spatially-explicit mosquito populations. (2018). doi:10.1101/350488
2. Otero, M., Solari, H. G. & Schweigmann, N. A Stochastic Population Dynamics Model for Aedes Aegypti: Formulation and Application to a City with Temperate Climate. *Bull. Math. Biol.* **68,** 1945–1974 (2006).
3. Christophers, S. & Others. Aedes aegypti (L.) the yellow fever mosquito: its life history, bionomics and structure. *Aëdes aegypti (L.) the Yellow Fever Mosquito: its Life History, Bionomics and Structure.* (1960).
4. Simoy, M. I., Simoy, M. V. & Canziani, G. A. The effect of temperature on the population dynamics of Aedes aegypti. *Ecol. Modell.* **314,** 100–110 (2015).
5. Focks, D. A., Haile, D. G., Daniels, E. & Mount, G. A. Dynamic life table model for Aedes aegypti (Diptera: Culicidae): analysis of the literature and model development. *J. Med. Entomol.* **30,** 1003–1017 (1993).
6. Horsfall, W. R. *Mosquitoes: Their Bionomics and Relation to Disease*. (1972).
7. Fay, R. W. The biology and bionomics of Aedes aegypti in the laboratory. *Mosq. News* **24,** 300–308 (1964).
8. Carvalho, D. O. *et al.* Suppression of a Field Population of Aedes aegypti in Brazil by Sustained Release of Transgenic Male Mosquitoes. *PLoS Negl. Trop. Dis.* **9,** e0003864 (2015).
9. Yamada, H., Parker, A. G., Oliva, C. F., Balestrino, F. & Gilles, J. R. L. X-ray-induced sterility in Aedes albopictus (Diptera: Culicidae) and male longevity following irradiation. *J. Med. Entomol.* **51,** 811–816 (2014).
10. Zhang, D., Zheng, X., Xi, Z., Bourtzis, K. & Gilles, J. R. L. ombining the sterile insect technique with the incompatible insect technique: I-impact of wolbachia infection on the fitness of triple- and double-infected strains of Aedes albopictus. *PLoS One* **10,** e0121126 (2015).
11. Bellini, R. *et al.* Mating competitiveness of Aedes albopictus radio-sterilized males in large enclosures exposed to natural conditions. *J. Med. Entomol.* **50,** 94–102 (2013).
12. Atyame, C. M. *et al.* Comparison of Irradiation and Wolbachia Based Approaches for Sterile-Male Strategies Targeting Aedes albopictus. *PLoS One* **11,** e0146834 (2016).
13. Massonnet-Bruneel, B. *et al.* Fitness of transgenic mosquito Aedes aegypti males carrying a dominant lethal genetic system. *PLoS One* **8,** e62711 (2013).
14. Harris, A. F. *et al.* Field performance of engineered male mosquitoes. *Nat. Biotechnol.* **29,** 1034–1037 (2011).
