# Supplementary material for: Transforming insect population control with precision guided sterile males with demonstration in flies
Source: Nat Commun. 2019 Jan 8;10:84. doi: 10.1038/s41467-018-07964-7 (PMC6325135; doi:10.1038/s41467-018-07964-7)
Supplement: Supplementary file 2 — Reporting Summary [file 41467_2018_7964_MOESM2_ESM.pdf]

natureresearch

Corresponding author(s): DBPR NCOMMS-18-24347A

Reporting Summary

Nature Research wishes to improve the reproducibility of the work that we publish. This form provides structure for consistency and transparency in reporting. For further information on Nature Research policies, see Authors & Referees and the Editorial Policy Checklist.

Statistical parameters

When statistical analyses are reported, confirm that the following items are present in the relevant location (e.g. figure legend, table legend, main text, or Methods section).

n/a	Confirmed	
☐	☒	The exact sample size (n) for each experimental group/condition, given as a discrete number and unit of measurement
☐	☒	An indication of whether measurements were taken from distinct samples or whether the same sample was measured repeatedly
☐	☒	The statistical test(s) used AND whether they are one- or two-sided<br>*Only common tests should be described solely by name; describe more complex techniques in the Methods section.*
☒	☐	A description of all covariates tested
☐	☒	A description of any assumptions or corrections, such as tests of normality and adjustment for multiple comparisons
☐	☒	A full description of the statistics including central tendency (e.g. means) or other basic estimates (e.g. regression coefficient) AND variation (e.g. standard deviation) or associated estimates of uncertainty (e.g. confidence intervals)
☐	☒	For null hypothesis testing, the test statistic (e.g. F, t, r) with confidence intervals, effect sizes, degrees of freedom and P value noted<br>*Give P values as exact values whenever suitable.*
☒	☐	For Bayesian analysis, information on the choice of priors and Markov chain Monte Carlo settings
☒	☐	For hierarchical and complex designs, identification of the appropriate level for tests and full reporting of outcomes
☐	☒	Estimates of effect sizes (e.g. Cohen's d, Pearson's r), indicating how they were calculated
☐	☒	Clearly defined error bars<br>*State explicitly what error bars represent (e.g. SD, SE, CI)*

Our web collection on statistics for biologists may be useful.

Software and code

Policy information about availability of computer code

Data collection	no software was used for data collection. We simulated release schemes for each using the MGDrivE simulation framework 43 (https://marshalllab.github.io/MGDrivE/).
Data analysis	JMP 8.0.2 by SAS Institute Inc.; Pearson's Chi-squared tests for contingency tables; Non-Parametric Maximum Likelihood Estimates (NPMLE) of the survival curves and Sun's generalization of the log-rank test was calculated in the R package "interval": http://www.jstatsoft.org/v36/ i02/.

For manuscripts utilizing custom algorithms or software that are central to the research but not yet described in published literature, software must be made available to editors/reviewers upon request. We strongly encourage code deposition in a community repository (e.g. GitHub). See the Nature Research guidelines for submitting code & software for further information.

Data

Policy information about availability of data

All manuscripts must include a data availability statement. This statement should provide the following information, where applicable:

- Accession codes, unique identifiers, or web links for publicly available datasets
- A list of figures that have associated raw data
- A description of any restrictions on data availability

The data supporting the findings of this study are available within the paper and its supplementary information files. Complete annotated plasmid sequences and plasmid DNA are publically available for order at Addgene. Transgenic flies have been made available for order from Bloomington Drosophila stock center. Accession codes for deposited plasmids and Drosophila stocks are listed in Supplementary Fig. 1.

Field-specific reporting

Please select the best fit for your research. If you are not sure, read the appropriate sections before making your selection.

☒ Life sciences ☐ Behavioural & social sciences ☐ Ecological, evolutionary & environmental sciences

For a reference copy of the document with all sections, see nature.com/authors/policies/ReportingSummary-flat.pdf

Life sciences study design

All studies must disclose on these points even when the disclosure is negative.

Sample size	The key data are raw counts of three different sexes in F1 progeny: male, female, and intersex. We scored at least 100-150 of F1 progeny flies for each replicate cross. To estimate egg hatching rate we counted at least 200 eggs for each replicate. To genotype targeted loci, at least two males and two females or intersexes were sequenced for each cross combination.
Data exclusions	No data were excluded.
Replication	Each experimental cross was repeated at least 3 times using 10 females and 10 males, in each crossing direction (maternal or paternal Cas9). The crosses of double gRNAs (dgRNAs) and different Cas9 lines (homozygous or heterozygous) were repeated at least four times in each direction. (N) is the number of true biological replicates, and (n) is the number of screened progeny. All raw data are presented in the supplementary tables. Five replicates were used for competition and longevity of pgSIT males.
Randomization	Each test cross was repeated in both directions (paternal & maternal Cas9 gene). Each important reciprocal cross (dgRNAs X Cas9) was set up and scored by three investigators (two investigators did it blindly) using virgin flies collected from aliquots of established transgenic lines. Flies used for sequencing and anatomy analysis were collected randomly, and we sampled these flies from separate progenies of different parents.
Blinding	Two investigators did crosses between dgRNAs and Cas9 lines, and F1 progeny analysis (counting and scoring females, males, and intersexes) blindly. dgRNA and Cas9 lines had plasmid codes instead of names, and thus both investigators did not know what sex ratios in F1 progeny they were going to get.

Reporting for specific materials, systems and methods

Materials & experimental systems

n/a	Involved in the study	
☒	☐	Unique biological materials
☒	☐	Antibodies
☒	☐	Eukaryotic cell lines
☒	☐	Palaeontology
☐	☒	Animals and other organisms
☒	☐	Human research participants

Methods

n/a	Involved in the study	
☒	☐	ChIP-seq
☒	☐	Flow cytometry
☒	☐	MRI-based neuroimaging

Animals and other organisms

Policy information about studies involving animals; ARRIVE guidelines recommended for reporting animal research

Laboratory animals	Drosophila melanogaster W[1118] and many balancer lines from Drosophila Bloomington Stock Center (#9750, #8621, #24486,

Laboratory animals	etc.), and many transgenic lines established in the study
Wild animals	none
Field-collected samples	none
